# Data mining of molecular dynamics data reveals Li diffusion characteristics in garnet Li_7_La_3_Zr_2_O_12_

**DOI:** 10.1038/srep40769

**Published:** 2017-01-17

**Authors:** Chi Chen, Ziheng Lu, Francesco Ciucci

**Affiliations:** 1 Department of Mechanical and Aerospace Engineering, The Hong Kong University of Science and Technology, Hong Kong, China; 2 Department of Chemical and Biomolecular Engineering, The Hong Kong University of Science and Technology, Hong Kong, China

## Abstract

Understanding Li diffusion in solid conductors is essential for the next generation Li batteries. Here we show that density-based clustering of the trajectories computed using molecular dynamics simulations helps elucidate the Li diffusion mechanism within the Li_7_La_3_Zr_2_O_12_ (LLZO) crystal lattice. This unsupervised learning method recognizes lattice sites, is able to give the site type, and can identify Li hopping events. Results show that, while the cubic LLZO has a much higher hopping rate compared to its tetragonal counterpart, most of the Li hops in the cubic LLZO do not contribute to the diffusivity due to the dominance of back-and-forth type jumps. The hopping analysis and local Li configuration statistics give evidence that Li diffusivity in cubic LLZO is limited by the low vacancy concentration. The hopping statistics also shows uncorrelated Poisson-like diffusion for Li in the cubic LLZO, and correlated diffusion for Li in the tetragonal LLZO in the temporal scale. Further analysis of the spatio-temporal correlation using site-to-site mutual information confirms the weak site dependence of Li diffusion in the cubic LLZO as the origin for the uncorrelated diffusion. This work puts forward a perspective on combining machine learning and information theory to interpret results of molecular dynamics simulations.

Making new Li batteries with higher capacity, better durability, and safety has been the object of a worldwide research effort since 1990s. In particular, replacing the organic electrolyte with a stable solid alternative has attracted tremendous interests from both industry and academia. Shifting from liquid electrolytes to solid electrolytes in Li battery potentially enables the use of Li metal as the anode, allows the application of high voltage cathodes, extends the lifetime, and greatly improves the safety^1^,^2^,^3^. Among the available solid conductors, inorganic Li oxides have raised broad attention as potential substitutes for the conventional liquid electrolytes. While the research efforts continue increase, only a few materials are relatively stable and are capable of sustaining a Li conductivity that is close to that of liquid electrolytes, i.e., 10^–1^~10^−3^ S cm^−1^ at room temperature (RT). Promising candidates include NASICON-type phosphates^4^, perovskite La_2/3-x_Li_3x_TiO_3_^5^, anti-perovskite Li_3_ClO^6^, Li_7_P_3_S_11_^7^,^8^, Li-β-alumina^9^, Li_3_N^10^, Li_4_SiO_4_^11^, Li_10_GeP_2_S_12_^12^,^13^, LiPON^14^, the garnet family^15^,^16^ and the recent Li_9.6_P_3_S_12_ and Li_9.54_Si_1.74_P_1.44_S_11.7_Cl_0.3_^17^, only to name a few^18^.

Within the Li-conducting garnet family, Li_x_La_3_M_2_O_12_ (x = 5 for M = Nb, Ta or x = 7 for M = Zr)^19^ has attracted extensive attention due to its relatively high Li conductivity at RT, high stability with respect to Li metal, compatibility with high voltage cathode materials^19^, and stability with water^20^. Among these garnet oxides, Li-stuffed Li_7_La_3_Zr_2_O_12_ (LLZO)^16^ holds great promise but also raises intriguing questions because it has two polymorphs that show vastly different Li conductivity. In the modeling community, various groups have been working on interpreting the properties of LLZO^21^,^22^,^23^,^24^,^25^,^26^. The experimentally observed lower Li conductivity in the tetragonal phase has been attributed to the Li vacancy ordering therein, whereas ordering does not occur in the cubic counterpart. From *ab inito* molecular dynamics (AIMD), Jalem *et al*. found synchronous Li movement in the cubic phase and an unstable 24d tetrahedral sites may trigger the hopping events, which eventually contributed to a high Li conductivity, i.e., 10^−4^ S/cm at 300 K^24^. In contrast, Meier *et al*.^27^ discovered, from molecular dynamics (MD) simulations, that while synchronous collective Li migration takes place in the tetragonal phase, the cubic phase is characterized by asynchronous hops. Later research by Burbano *et al*.^28^ showed that the slow cyclic-type Li movements in the tetragonal LLZO explained the low conductivity. One reason for this discrepancy may lie in the analysis of the Li hops. While the study of a relatively small system in AIMD simulations allows for the tracking of the atom jumps with ease, the conclusions may not be statistically accurate due to the small size and the limited number of Li hops. On the other hand, the large trajectory dataset obtained from classical MD simulations enables a credible statistical analysis but at the same time its massive size is a barrier to obtaining insight from the data. In addition, the spatial distribution of lithium around its equilibrium lattice sites adds to the difficulty since the identification of the Li jumps would require an accurate spatial definition of Li sites. Previous works have used spherical regions or polyhedral constructions to assign particles to given sites, where the site location was given *a priori*^29^,^30^,^31^. Other works borrowed ideas from computer science and leveraged the crystallographic information by performing k-means clustering to link particles to specific sites^32^,^33^,^34^. However, the k-means clustering is known to be NP-hard and a large dataset would require a good initial guess of centers. On the other hand, despite that they are relatively easy implementations, all these methods are only able to separate datasets with convex shape, i.e., for each data point pair, the data point that lies on the straight line connecting the data pair is also within the shape region. In ionic systems, if ions stay stationary, the crystallographic sites of these ions are defined on their locations. However, at finite temperatures, the thermal effects trigger faster vibration and movement of the ions. In this scenario, the crystallographic sites could be defined by aggregating the ion trajectories over time and then assigning the regions with higher density as the crystallographic sites. In other words, the continuous movement of particles allows one to define the nuclear density where the low density regions can be representative of site separation. In this work, we develop a novel density-based clustering of trajectories (DCT) method that learns the site information from the MD simulation trajectories. This method calculates the nuclear density from the trajectories and learns information from the density.

## Results and Discussion

**Figure 1:**
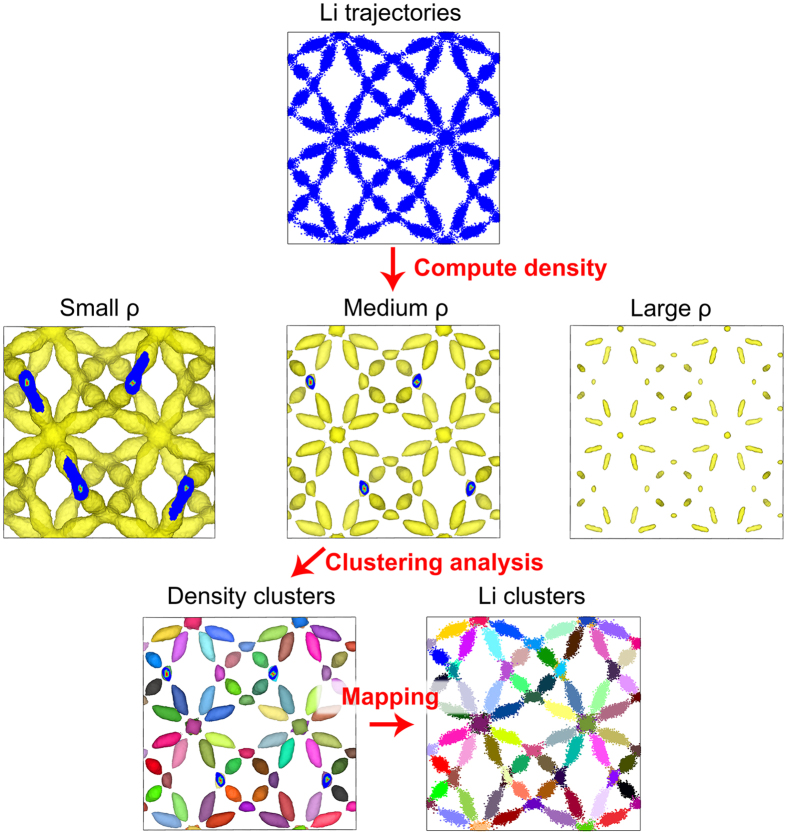
The clustering scheme illustrated by the cubic LLZO simulated at 400 K. Only a 1 × 1 × 1 cell is shown for clearly vision inspection.

The DCT method developed in this article is based on the observation that ionic diffusive events in the crystal lattice are relative infrequent relative to the usual simulation scales. In addition, the ions mostly oscillate around their equilibrium lattice site, leading to high density therein as depicted in Fig. 1. This insight leads to the development of the following algorithm: first, the Li nuclear density is estimated from all Li ion trajectories by binning the Li positions to cubic voxels of side length *dr.* We note that by taking *dr* = 1 Å the density pattern from the simulation matches well its experimental counterpart as obtained from diffraction^35^. Second, we select a local density threshold *ρ* to separate the various spatial regions. As shown in Fig. 1, a small *ρ* results in connected density, which cannot be used to differentiate the Li sites. On the other hand, when it is too large, the corresponding clusters are too small causing potential loss of information. We suggest that one could choose an intermediate *ρ* such that


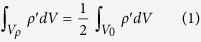


where *V*_0_ is the entire domain of the simulation and *V*_*ρ*_ is the region with density greater than *ρ*, i.e., 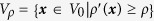. We shall call *V*_*ρ*_ the core domain and the corresponding voxels the core voxels. The voxels, whose density are nonzero and less than *ρ*, are named noise voxels. Third, we use a breadth-first search algorithm to find core voxels adjacent to interested core voxels and therefore to identify the voxel clusters. Starting from one core voxel, the breadth-first search method finds all the neighboring core voxels and then for each of them, the neighboring search is repeated until no connected core voxels are further found, see Scheme S1 for illustration. Lastly, in the mapping step, a reverse density calculation that map the density to the particles is performed to assign cluster labels of the voxel to the particles therein.

The DBSCAN algorithm developed by Kriegel and co-workers^36^ shares similar characteristics as our method, such as the ability to cluster the data based on the density and the ability to automatically recognize non-convex spatial patterns. However, the DBSCAN does not compute the density directly, but relies on calculating the distance between every data entry. This is advantageous when dealing with higher dimensional data on small datasets. In contrast, the data size of MD trajectories is typically large while the dimensionality of the particle trajectory is relatively low (3 dimensions). While DBSCAN requires the computation of all the pair distances (i.e. if the simulation is characterized by *N* atoms and *T* snapshots then it requires (*NT*)^2^ operations), DCT relies on the computation of density and the neighboring search is done by indexing, requiring *M* calculations for a density map with *M* voxels. In addition, the nuclear density computation is ubiquitous in molecular dynamics simulations as a way to assist the interpretation of particle transport mechanism^37^,^38^,^39^,^40^,^41^. The proposed method therefore can be used in conjunction with the exploratory data analysis of the nuclear density pattern. Lastly, the clustering analysis yields two time series. The first time series consists in the site occupancy of all available sites with time. The second time series tracks how each Li’s site changes with time. In MD analysis, velocity autocorrelation functions and Van Hove correlation functions are widely used techniques^42^. While both methods can be used to reduce the data and to categorize particles, any local effects, such as the impact of the neighboring cationic environments, are lost in the analysis. The DCT framework complements these methods by allowing the tracking of individual particles, and the determination of the overall particle dynamics.

The clustering results on a 2 × 2 × 2 supercell give a total number of 576 clusters (all available sites) for the cubic LLZO (c-LLZO) in the whole temperature range, and 448 clusters for the tetragonal LLZO (t-LLZO) from 300 K to 900 K and 576 cluster for temperatures greater than 900 K. The change of cluster number of t-LLZO at 900 K is accompanied by Li diffusivity change as t-LLZO starts to be similar to c-LLZO above 900 K, see Figure S1. The abrupt change in the total number of Li clusters and diffusivity in t-LLZO suggests that certain transition occurs at a temperature between 900 K and 1000 K^43^. In c-LLZO we did not find the splitting of 48 g sites into 96 h sites in c-LLZO since the two sites are closely connected.

As shown in Fig. 1, since we have the site attributes for all Li ions at all time steps, we can use this to derive the site characteristics. Even without crystallographic information, the cluster analysis of the local Li density detects two types of sites in c-LLZO (i.e. 48 g and 24d sites) and three occupied site types in t-LLZO (i.e., 8a, 16f and 32e sites) as shown in Section S2. The determination of crystallographic sites enables the validation of the simulations against experiments and could be in principle used to detect phase transitions. In addition, the site occupancy can also be obtained as discussed in Section S3. Generally, Li vacancy clusters are rarely found and the most frequent local configurations of Li occupancy are T_04_, T_13_ and O_11_ as shown in Figure S6, where the upper case letter indicates the center site (Td or Oh), the first index is the number of Li at the center site and the second index is the total number of Li nearest neighbors.

The DCT allows us to obtain the statistics of the Li jumps, which, as noted above, is naturally encoded in a time series obtained by associating every Li to an identified cluster at a given time step. The hopping rate in c-LLZO is greater than that in t-LLZO, see Figure S8. The activation barrier energy is also different within the two phases, as shown in Figure S9, where the barrier of t-LLZO is higher.

**Figure 2:**
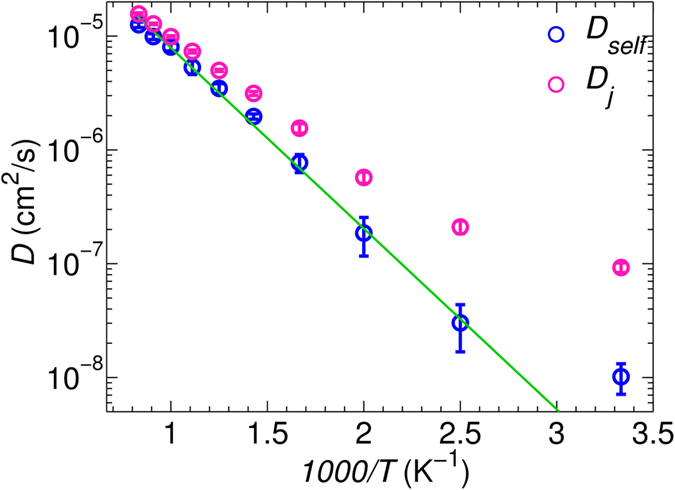
Diffusivities computed from hopping statistics *D*_jump_ vs diffusivities computed from fitting MSDs. The red line indicates the least square fit of the simulation data against 
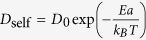
. The parameters are *D*_0_ = 3.06 × 10^−4^ cm^2^s^−1^ and *Ea* = 0.31 eV.

We notice that in c-LLZO, despite the high jump rate, it is highly probable that Li returns to the original site after hopping to the neighboring site as shown in Figure S10. Since the net displacement of the Li is zero in the back-and-forth jumps, such events do not contribute to the Li diffusivity. To verify our assumption, we computed the jump diffusivity of Li, *D*_jump_, from the jump statistics by the following formula^29^


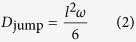


where *l* is the distance between neighboring site centers, calculated from the average Li coordinates within the given center, and *ω* is the Li hopping frequency. We filter out the back-and-forth Li hops and compute the jump diffusivity *D*_jump_. The comparison between *D*_jump_ and *D*_self_, is shown in Fig. 2. Reasonable agreement is reached as a result of filtering out the back-and-forth Li hops. Without filtering, however, the jump diffusivity is much higher, as shown in Figure S12d. The difference is even more prominent at lower temperature.

This result also supports our previous conjecture that, since T_04_ and T_13_ configurations are the most frequent, Li diffusion is relatively short-ranged because continuously open channels are missing. In other words, the analysis suggests that one bottleneck to higher diffusion rate is the relatively low vacancy concentration. If more vacancies are created, the total number of back-and-forth Li hops could be reduced, thereby resulting in more effective Li diffusional rates. Therefore, it is expected that doping the LLZO with higher valence cations increases the Li vacancy concentration and enhances Li self-diffusion. This has been observed if LLZO is doped with, for example, penta-valent elements Ta^5+^ and Nb^5+^ 23,44,45,46 or if Li is partially substituted with Al^3+^ and Ga^3+ ^47,48,49. As shown in Figure S13, an increase of diffusivity is observed if the vacancy concentration increases, and this trend continues until the composition reaches Li_5.3_La_3_Zr_2_O_12_.

**Figure 3:**
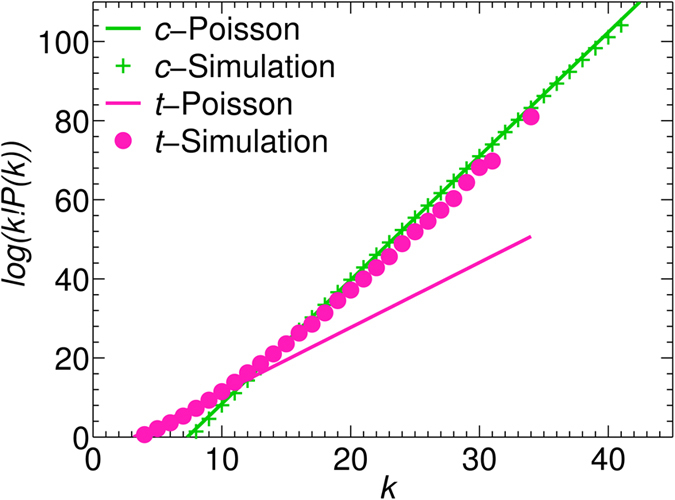
Li hopping statistics from the simulation, compared to ideal Poisson process. *k* is the jump event number per unit cell in 1 ps.

The intriguing Li jump characteristics calls for a more detailed study, especially on the correlation between Li jumps and Li sites both in time and space. The nature of the temporal stochastic process can be studied by benchmarking the probability *P*(*k*) of observing k simultaneous jumps within 0.1 ps against the Poisson distribution 
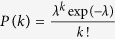
50. Interestingly, the Li hopping in c-LLZO follows a Poisson distribution (see also Figure S20), while t-LLZO deviates substantially from it as shown in Fig. 3 for simulations at 900 K (data at other temperatures are shown in Figure S14). This correlated jump behavior in t-LLZO is consistent with previous findings by Meier *et al*.^27^ and Burbano *et al*.^28^.

**Figure 4:**
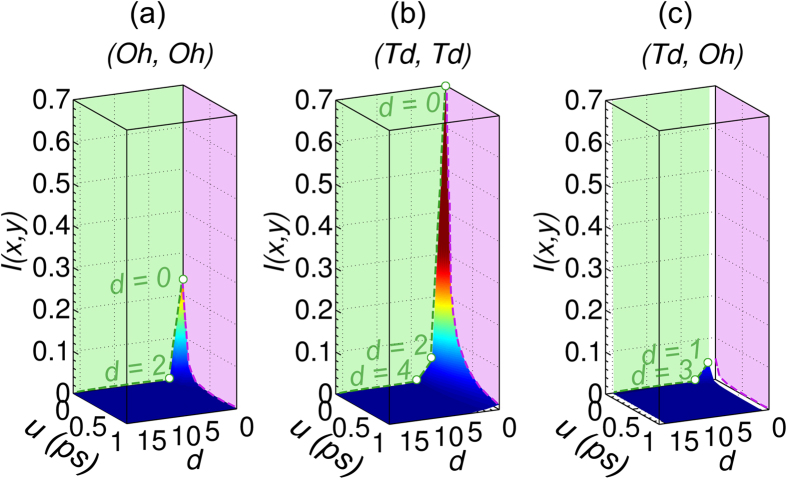
Site mutual information versus geometrical distance d and output time step delay u of c-LLZO at 1200 K for (Oh, Oh) (**a**), (Td, Td) (**b**) and (Td, Oh) (**c**).

In c-LLZO, while the aggregate behavior of Li hopping appears to be qualitatively Poissonian in the temporal scale, studying the site-to-site interaction may provide further spatial scale information. In order to clarify the spatial correlation among Li sites, we calculated the site mutual information for all site combinations as defined by ref. 51.


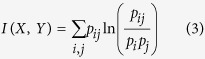


where *X* and *Y* are the random variables denoting corresponding sites and *p*_*ij*_ is the *p*robability of the (*X, Y*) to be in occupancy state (*i, j*), where *i* and *j* could be 0 or 1, e.g., *p*_01_ is the probability that site *X* is unoccupied and site *Y* is occupied. Detailed explanation is provided in Section S5. We note that such mutual information is the Kullback-Leibler distance of the individual site occupancy distributions, implying that it measures the amount of information into *X* is contained in *Y*, i.e., the site dependence. It is important to note that *I*(*X, Y*) > 0 indicates dependence between *X* and *Y,* and *I*(*X, Y*) = 0 if and only if *X* and *Y* are independent. Furthermore, the mutual information is positively related to the correlation. In the special case where *X* and *Y* are Gaussian random variables, *I*(*X, Y*) is related to the bivariate correlation *ρ*_*b*_(*X, Y*) following the formula 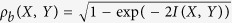
52. In addition to *I*(*X, Y*), we also computed the delayed mutual information defined as 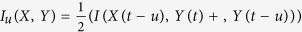
53,54. All data are presented as a function of time delay and static site distance, i.e., the number of hops needed to go from one site to the another as computed by Dijkstra’s algorithm^55^, see Fig. 4. Other details are presented in Section S5.

**Figure 5:**
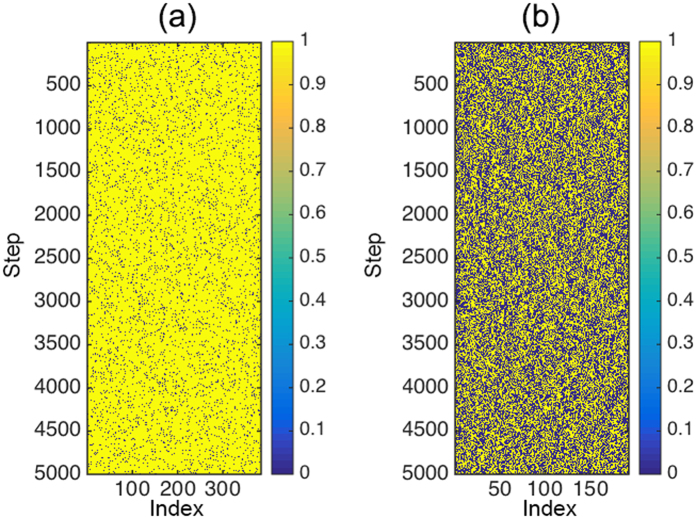
The site occupancy of 384 Oh sites and 192 Td sites for 500 ps (5000 output time steps at 1200 K.

The value of *I*_*u*,*d*_(*X, Y*) when *u* = *d* = 0 corresponds to the entropy. Due to an occupancy close to 0.5 for the Td sites and greater randomness than the Oh sites, see Fig. 5, the entropy of Td sites is higher than that of the Oh sites, as shown in Fig. 4.

The autoinformation, that is *I*_*u*,*d*_(*X, Y*) for *u* > 0 and *d* = 0, measures the autocorrelation of a given *X* site. As a consequence of occupancy and in spite of considerable site randomness, the Td sites are slightly autocorrelated as shown in Fig. 4b and, for T = 1200 K, the autocorrelation time is on the order of 1 ps. We can also show the mutual information as a function of distance. Td sites seem to have a weak long-ranged spatial correlation which is greater than the Oh-Oh and Td-Oh. 1D plots of mutual information *I*_*u*,*d*_(*X, Y*) versus *d* for fixed *u* are shown in Figure S17.

Previous analysis on the site occupancy in Section S3 demonstrated that certain neighboring occupancy patterns, such as O_11_, occurred at a higher probability than others. Surprisingly, this does not mean that the neighboring Oh and Td site are highly correlated. On the contrary, the neighboring sites show decreased correlation in comparison to the Td-Td and Oh-Oh interactions at second nearest neighbors, as evidenced by the low mutual information between Oh and Td in Fig. 4c. Overall, the spatial correlation is weak and short-ranged, confirming the observed uncorrelated Poissonian type of diffusion. Mutual information results at other temperatures also show weak correlation, as shown in Figure S18.

## Conclusions

Li diffusion in the cubic and tetragonal phase LLZO is studied by MD simulations. We develop a density-based clustering method to obtain the Li site shape, type and occupation information, and Li jump statistics. The results show that in c-LLZO, the Li occupancy is much higher at the Oh sites than the Td sites, and t-LLZO has an occupancy of 1 for three types of sites. Our study also shows that at RT the Li jumps in c-LLZO consist mostly of a back-and-forth type, and this type of jumps does not contribute to the effective diffusion. In line with the Li occupancy analysis, the reason for the back-and-forth jump is that when Li jump from an Oh to the neighboring empty Td site, the other diffusion paths are mostly blocked by the Li. Therefore, the main obstacle to Li diffusion in LLZO is the low vacancy concentration. Substituting cations with higher valence is an effective strategy to increase the vacancy concentration, as found in experimental observations. In addition, Li hops in c-LLZO are uncorrelated and fit well with the Poisson process while it deviates from the Poisson process in t-LLZO. Lastly, the information theoretic analysis of c-LLZO confirms that the low correlation between sites likely induces a Poissonian type of Li hopping. However, the Li sites still have some short-ranged time and space correlation and this correlation is stronger for the Td sites in comparison to the Oh sites.

## Methods

MD simulations were performed in LAMMPS^56^. The potential parameters, presented in Table S1, were fitted from DFT calculations by Klenk and Lai^43^, where the lattice parameters, phase transition temperatures, and Li diffusivities are benchmarked against previous experiments^57^. In order to simulate both the cubic and tetragonal phase of LLZO, and examine the difference in Li diffusion behaviors, we artificially imposed isotropic pressure and biaxial pressure respectively to constraint the lattice shape. For the cubic phase, since 2/9 of the Li sites are vacant, we generated randomly distributed Li vacancy positions and constructed 8 different vacancy configurations. The simulated trajectories from different configurations are collected together to yield the nuclear density estimate. The simulation temperatures ranged from 300 K to 1200 K with a 100 K interval. During each simulation, the temperature was first equilibrated at 1200 K and then cooled to the set temperature in the NPT ensemble employing a Noose-Hover thermostat and barostat with a damping parameter of 0.05 ps for the temperature and 0.25 ps for the pressure. Then the systems were equilibrated at the set temperature for 50 ps followed by the data collection run in NVT ensemble. For the cubic phase, the simulation time was 500 ps long and the trajectories were sampled every 0.1 ps. For the tetragonal phase, a longer 4 ns run was performed, where the same sampling rate was used. The 4 ns runs of tetragonal phase at low temperatures, especially at 300 K, may not be able to generate reliable diffusivity results due to the intrinsically slow Li movements. However, the simulation results have shown experimentally-consistent characteristics of the site information, such as the site types and multiplicity, and the low Li jump statistics, which are necessary in the current study as explained in the results section. For all simulations, a velocity Verlet algorithm with a 1 fs interval was chosen for the time-stepping method. For a system with extra Li vacancies that cause unbalanced charge, a uniform background charge is added, as implemented in LAMMPS.

## Additional Information

**How to cite this article**: Chen, C. *et al*. Data mining of molecular dynamics data reveals Li diffusion characteristics in garnet Li_7_La_3_Zr_2_O_12_. *Sci. Rep.* **7**, 40769; doi: 10.1038/srep40769 (2017).

**Publisher's note:** Springer Nature remains neutral with regard to jurisdictional claims in published maps and institutional affiliations.

## Supplementary Material

Supplementary Information
