# Supplementary material for: Data mining of molecular dynamics data reveals Li diffusion characteristics in garnet Li7La3Zr2O12
Source: Sci Rep. 2017 Jan 17;7:40769. doi: 10.1038/srep40769 (PMC5240091; doi:10.1038/srep40769)
Supplement: Supplementary Information [file srep40769-s1.doc]

**Electronic Supplementary Information**

**Data mining of molecular dynamics data reveals Li diffusion characteristics in garnet Li_7_La_3_Zr_2_O_12_**

**Chi Chen, ^a^ Ziheng Lu ^a^ and Francesco Ciucci ^a, b^, ***

*^a^ Department of Mechanical and Aerospace Engineering, The Hong Kong University of Science and Technology, Hong Kong, China. Prof. Francesco Ciucci (francesco.ciucci@ust.hk)*

*^b^ Department of Chemical and Biomolecular Engineering, The Hong Kong University of Science and Technology, Hong Kong, China*

1. **Potential models, algorithm explanation and basic results.**

*Potential model parameters and calculation methods*

The parameters for the Buckingham potential model is as follows

Table S1. Buckingham-type potential parameters used in this work.[^1^](#_ENREF_1)

|  | z (e) | A (eV) | ρ(Å) | C (eV Å^6^) |
| --- | --- | --- | --- | --- |
| Li---O | 1.00 | 1087.29 | 0.260 | 0.00 |
| La---O | 2.50 | 2075.26 | 0.326 | 23.25 |
| Zr---O | 2.65 | 1650.32 | 0.311 | 5.10 |
| O---O | -1.65 | 4870.00 | 0.267 | 77.00 |

*DCT*

*Scheme for the clustering analysis*


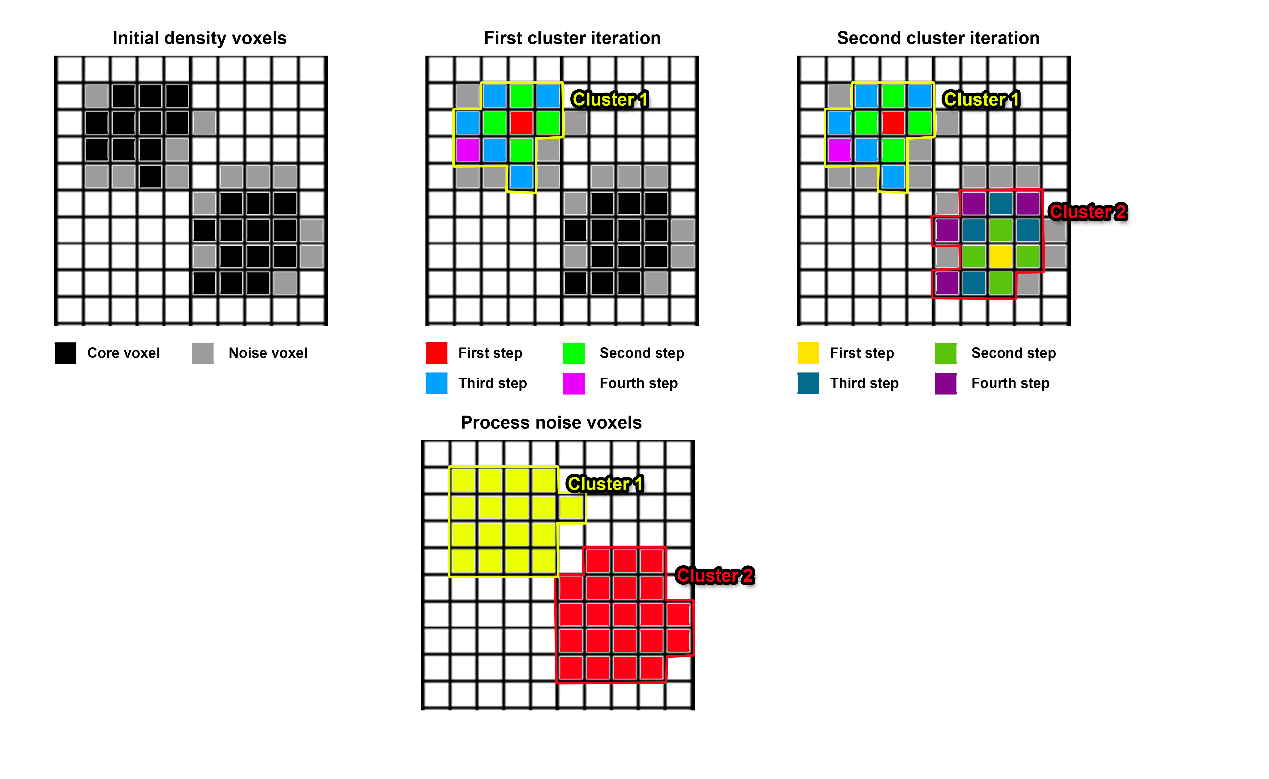


Scheme S1. A 2D example illustrating the clustering iteration steps.

*Diffusivity and site clustering results*

The comparison between the diffusivity of c-LLZO and t-LLZO is shown in Figure S1 below.

| (a) | (b) |
| --- | --- |
| 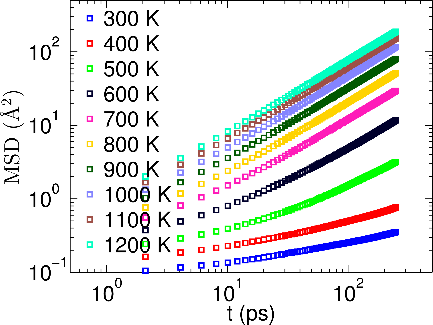 | 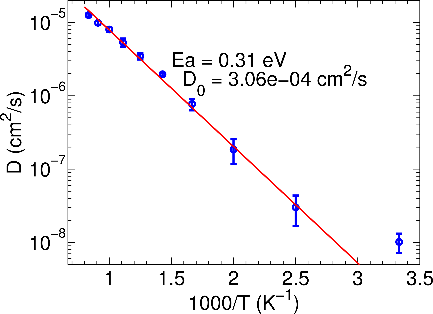 |
| (c) | (d) |
| 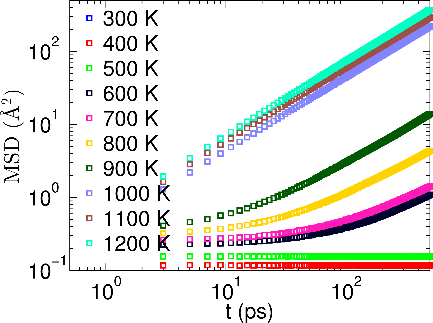 | 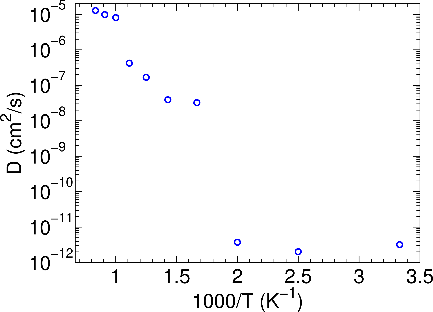 |

Figure S1. Calculated mean-squared displacements of c-LLZO (a) and t-LLZO (c), and diffusivities at various temperatures of c-LLZO (b) and t-LLZO (d).

The Li clusters are presented in Figure S2, where only a 1x1x1 cell is shown for simplicity and the Li ions are arbitrarily colored based on their specific cluster. As evident in the graphs, c-LLZO shows a continuous Li diffusion path while t-LLZO shows dense Li atoms only at specific sites. 48g and 24d sites in c-LLZO and 8a, 32g, 16f sites are also identified thanks to clustering. Moreover, diffusion occurs along a ring structure formed by 12 sites as shown in Figure 2c and d. In c-LLZO, these 12 sites consist of alternating 24d and 48g sites, while the empty 16e sites interrupt the ring structure in t-LLZO.

| (a) | (b) |
| --- | --- |
| 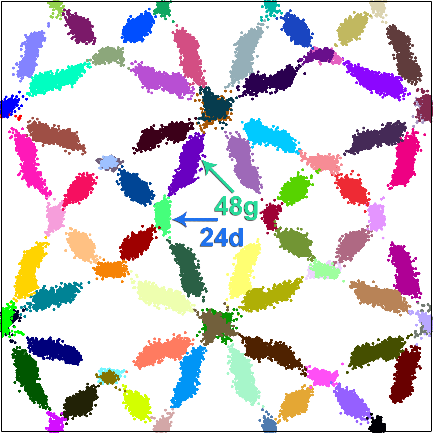 | 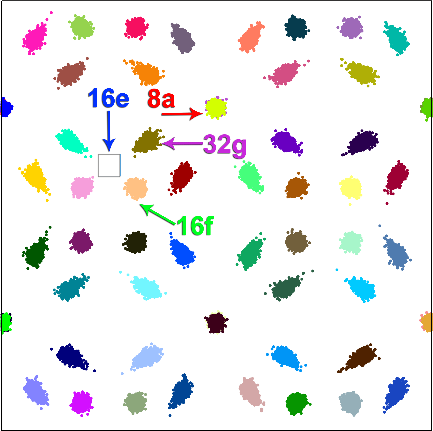 |
| (c) | (d) |
| 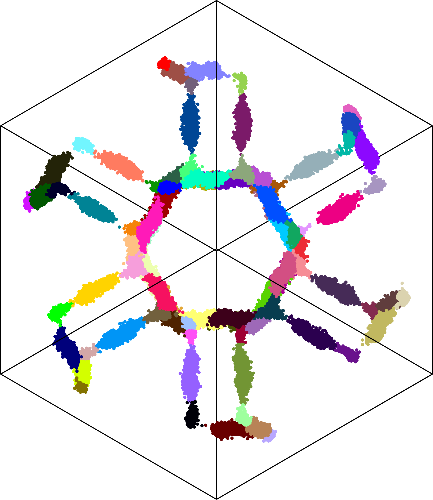 | 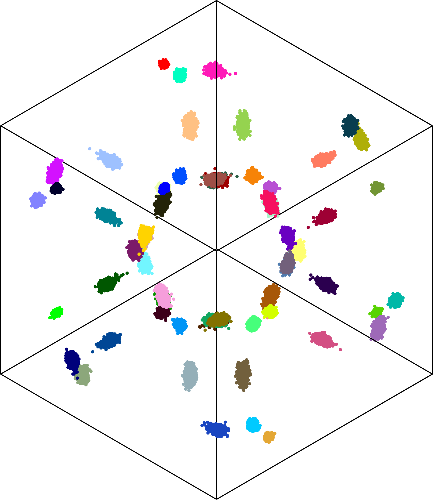 |

Figure S2. Li clusters at 300 K for 1x1x1 cell, (a) and (c) cubic phase viewed along –b axis and (b), (d) tetragonal phase viewed at isometric view.

**2. Geometrical features of sites revealed by clustering**

Principal component analysis (PCA) is used to study the shape of Li clusters in LLZO. Let
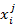
 denote the *i*-th coordinate of atom *j* (*i* = 1, 2, 3). Then the dimensional covariance matrix of the atom coordinates in cluster c can be computed as

| 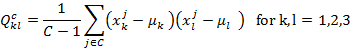 | (1) |
| --- | --- |

where *c* indicates the *c*-th cluster, and the corresponding upper case is the number of Li in this cluster.
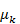
 is the *k*-th component of the mean coordinates. The summation is performed only in cluster c. PCA is then applied to the matrix
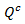
, and the corresponding eigen values
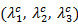
 are indicative of the geometric shapes of the cluster
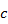
.

We plot the eigen values
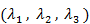
 of the all clusters (576 for the cubic and 448 for the tetragonal) in Figure S3. As shown therein, c-LLZO has two types of Li sites, indicated by the two eigen values regions and t-LLZO shows three types of sites, Figure S3b. Interestingly, the number of points in each one of two site types in the Figure S3a, matches well with the crystallography information; by counting the site number, we notice that the number of red colored points is 24 per unit cell and blue is 48 per unit cell, corresponding to the 24d and 48g site respectively. Therefore, the site type multiplicity is obtained from the clustering analysis even without the input from crystallography. Similarly, the three types of sites in the tetragonal phase give direct information of the 8a, 16f and 32g sites. We notice that the multiplicity of these sites are 8, 16 and 32 respectively, corresponding to 8a, 16f and 32g sites. In a word, the analysis enables the classification of the sites.

| (a) | (b) |
| --- | --- |
| 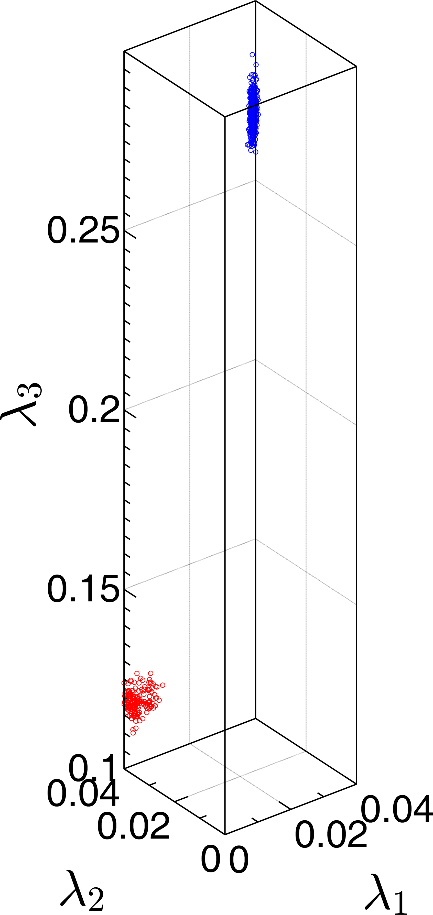 | 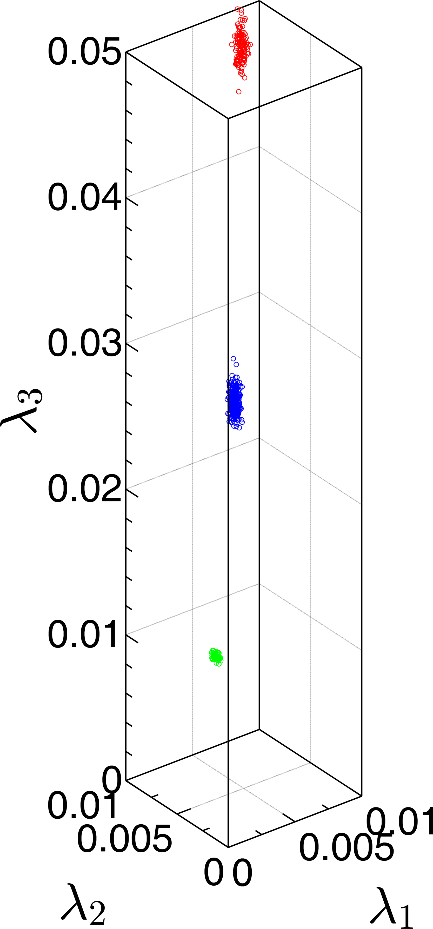 |

Figure S3. Eigen value points (
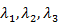
) for cluster covariance matrices of (a) c-LLZO at 1200 K and (b) t-LLZO at 300 K. The temperatures are chosen to represent the most stable structures.

Moreover, the eigen values are directly related to the shape and size of the site. In the cubic phase, one type of sites, marked in blue color in Figure S3a, shows clearly elongated shape, with the maximum eigen value much greater than the other two while the smaller two eigen values are almost equal. The other type of sites is smaller geometrically, as shown in red points in Figure S3a. Correspondingly, two examples of the corresponding two types of sites for the cubic phases are shown in Figure S4a and S4b. On the other hand, the plots of the eigen values for t-LLZO show a total of three types of sites. Representative site shapes are shown in Figure S4c, d and e.

| (a) |  | (b) |
| --- | --- | --- |
| 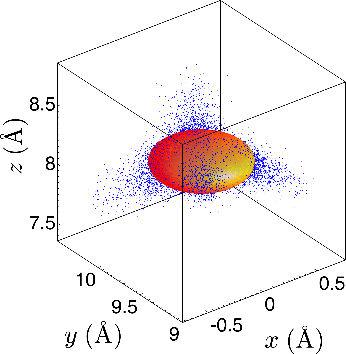 |  | 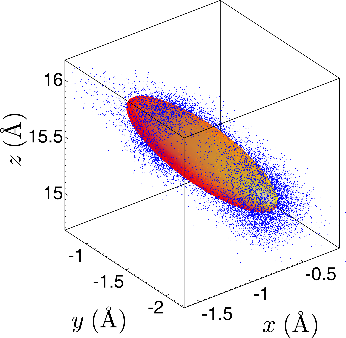 |
| (c) | (c) | (e) |
| 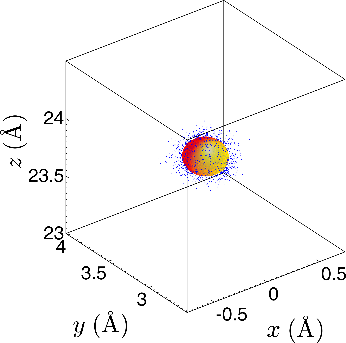 | 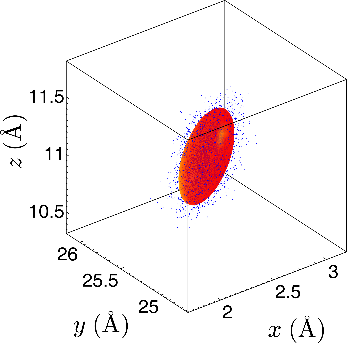 | 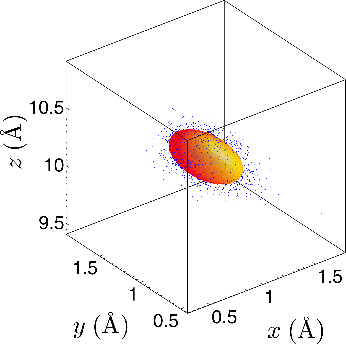 |

Figure S4. Shape difference of sites in 24d (a), 48g (b) in c-LLZO and 8a (c), 16f (d) and 32g (e) in t-LLZO.

The tetrahedral sites (Td) in both phases Figure S4a and c bear similar traits and the octahedral sites (Oh) show elongated shapes, Figure S4b, d and e. The geometric shapes suggest that the Oh sites, with elongated shape, mainly provide the conduction channel, while the Td sites work as the joints between these channels. With temperature change, the magnitude of vibration shall increase in both materials, leading to the increase of the corresponding eigen values, as shown in Figure S5.

| (a) |  | (b) |
| --- | --- | --- |
| 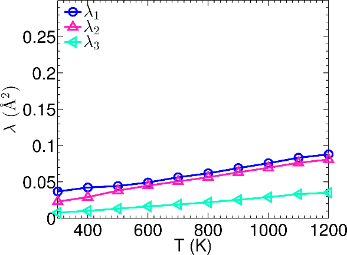 |  | 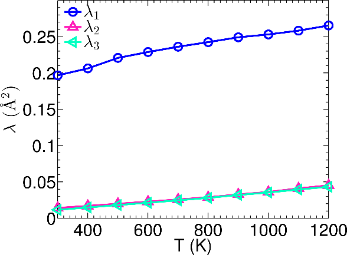 |
| (c) | (d) | (e) |
| 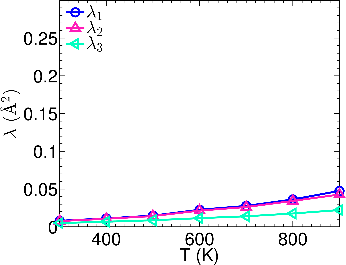 | 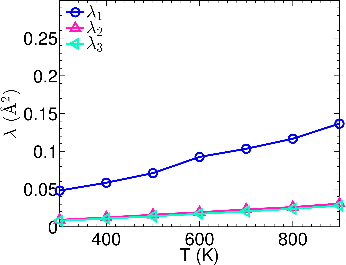 | 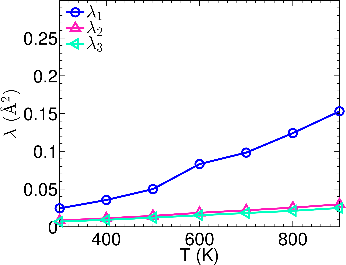 |

Figure S5. The ellipsoid size change, as represented by the eigen values of coordinate covariance matrix, with temperature for 24d (a) and 48g (b) sites in c-LLZO, and 8a (c), 16f (d) and 32g (e) sites in t-LLZO.

1. **Site occupancy analysis**

While higher temperature induces higher vibration and diffusion in the materials, it may also alter the Li site occupancy. It was generally accepted that Li vacancy ordering is the limiting factor that hinders the Li diffusion in t-LLZO. The Li vacancy ordering in c-LLZO seems controversial at the moment. We therefore study the distribution of Li distribution of vacancies in c-LLZO. Each Td site is connected to four Oh sites and each Oh site is adjacent to two Td sites. We indicate the occupancy of the centering site by the first subscript and the occupancy of the neighboring sites by the second subscript. For example T_10_ means the local configuration where the centering T site is occupied by Li and no Li is present at the neighboring two O sites [^2^](#_ENREF_2). In general, Li vacancy clusters are not favorable, as indicated by low values for T_00_ (pentamer vacancy cluster), T_01_ (tetramer), T_02_, O_00_ (trimer), T_03_, O_01_ (dimer vacancy cluster) within one unit cell, shown in Figure S4a and b. The results agree well with the calculations by Klenk et al. [^3^](#_ENREF_3). Overall, the most favorable Li configuration is an empty Td site with four occupied Oh sites, and an occupied Oh site with one neighboring filled Td site. This is equivalent to say that filled Td site tends to be surrounded by more Li atoms, while empty Td site can almost only tolerant no neighboring Li vacancies, as shown in Figure S6a. The Oh site centering configuration shows a nonlinear behavior on the other hand; when one Oh site is occupied by Li, the most probable configuration is one Li vacancy in the two neighbors, followed by two vacancies and lastly two occupied T site. Furthermore, with increasing temperature, the O_11_ occurrence decreases, balanced by an increasing of O_12_. Due to the higher occurrence of T_04_ and T_13_, one dominating transport pattern of Li may be the Li jump that alternate the local Li configuration between T_04_ and T_13_. However, this type of Li diffusion is extremely short-ranged since when Li hops from an Oh site to the Td site, changing the local structure from T_04_ to T_13_, the future paths are blocked by other Li, limiting the diffusion rate. This is also shown in the next nearest neighboring analysis Figure S6c and d, where O2_16_ sites are the most frequent configurations. Even Li at one O site is able to jump to the neighboring T site, the next jump to the NNN of the original Oh site is not possible due to the high chance of fully occupied NNN Oh sites.

| (a) | (b) |
| --- | --- |
| 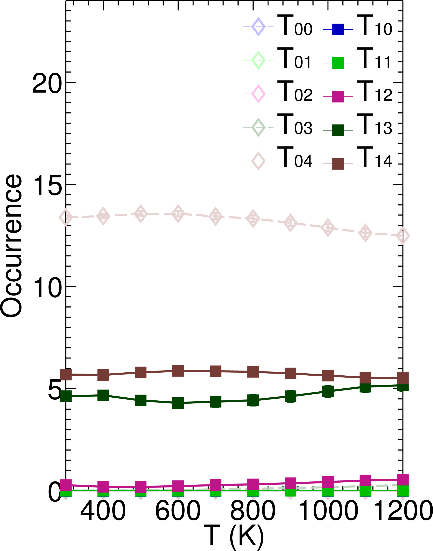 | 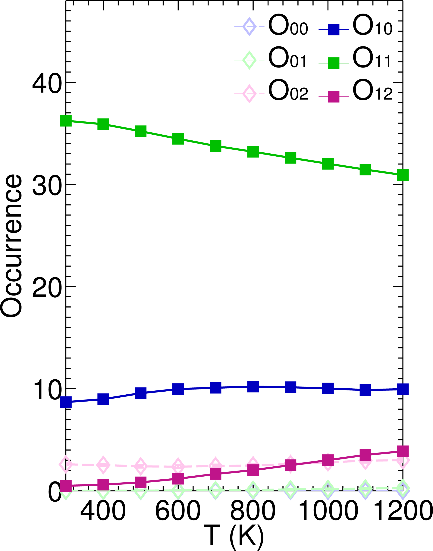 |
| (c) | (d) |
| 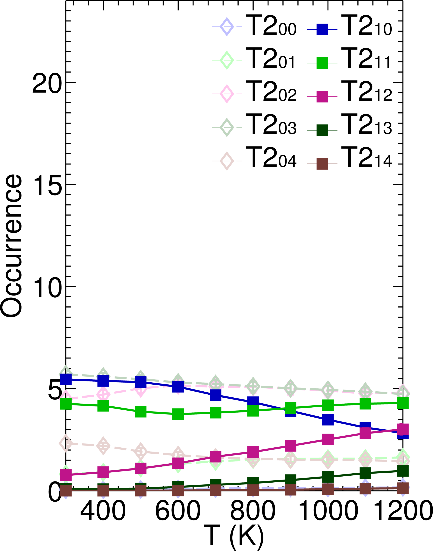 | 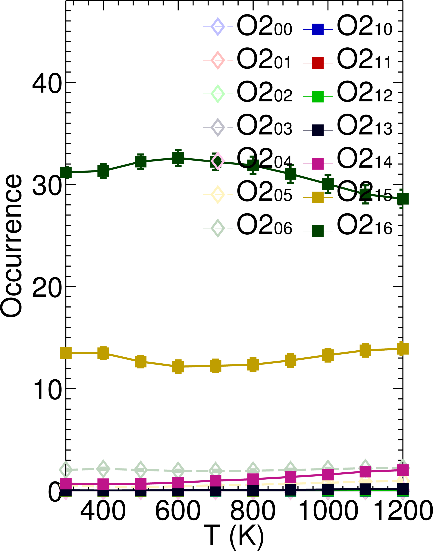 |

Figure S6. Occurrence of certain type of nearest neighbor configuration. Td and Oh indicate tetrahedral 24d and octahedral 48g site respectively. (a) and (b) show the nearest neighbor configurations, and (c) and (d) the next nearest neighbors. The first index in the subscript means the Li presence at the site and the second index indicates the number of Li at nearest neighbor/NNN. Note that each 48g site is connected by 2 24d sites and each 24d site by 4 48g sites. The NNNs of one Oh-48g site are 6 Oh-48g sites and of one Td-24d are 4 Td-24d sites.

We pursue the site connectivity and occupancy even further, beyond the nearest neighbors. From the site label and site connection information, we can construct a minimum path graph between any two sites using the Dijkstra's algorithm [^4^](#_ENREF_4), extending the local configuration analysis to a diffusion channel analysis. As shown earlier in Figure 2 in the main text, the basic diffusion unit of Li is formed by a 12-site ring structure. The analysis of a 2x2x2 cell shows 320 rings and the change of occupancy of one example is shown in the animation in supplementary information. We find that Li diffusion does not completely go over one whole ring structure, but rather they diffuse quite randomly. We clearly observe a higher Li occupancy of the octahedral 48g sites in the ring structure. The statistical information of site occupancy is shown in Figure S7a. The site occupancy seems not sensitive to the temperature, consistent with experimental observations [^5^](#_ENREF_5). In addition, the time-average occupancy of labeled sites is shown in Figure S7, signifying a larger scattering at lower temperature due to the low diffusion rate.

| (a) | |
| --- | --- |
| 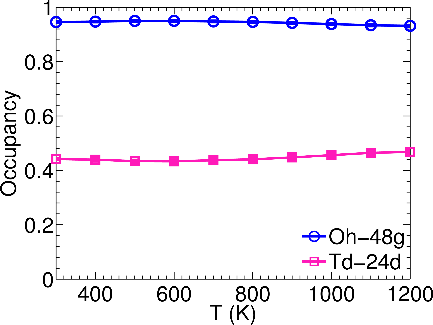 | |
| (b) | (c) |
| 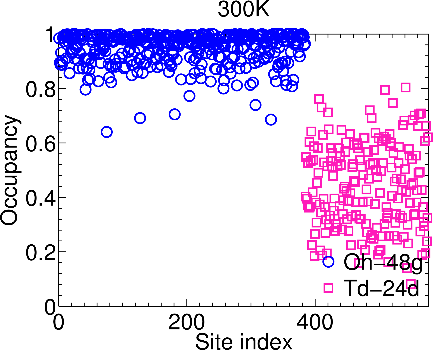 | 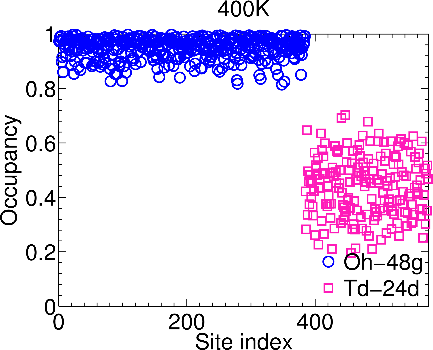 |
| (d) | (e) |
| 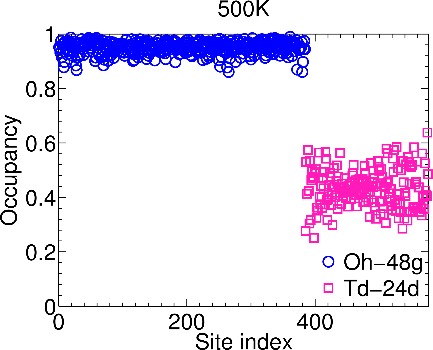 | 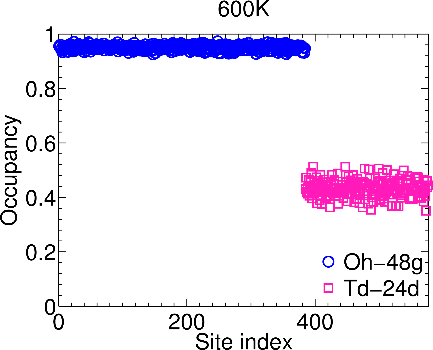 |
| (f) | (g) |
| 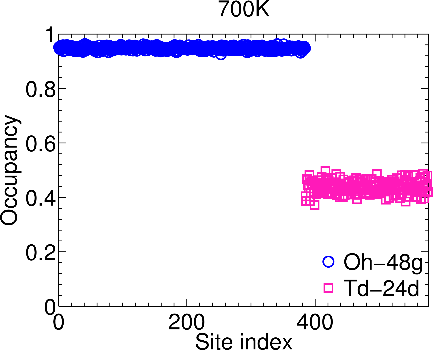 | 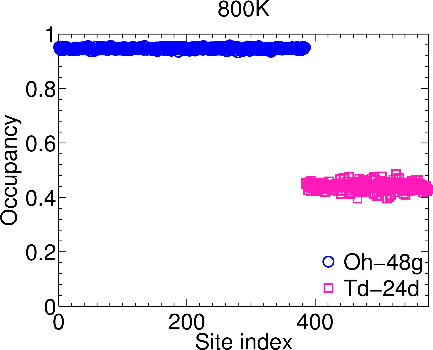 |
| (h) | (i) |
| 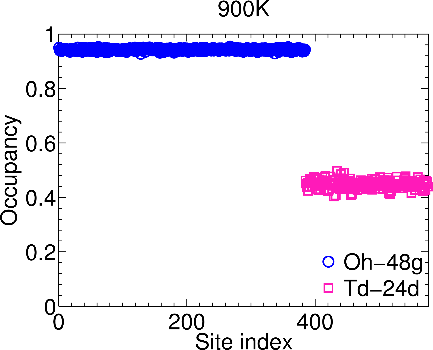 | 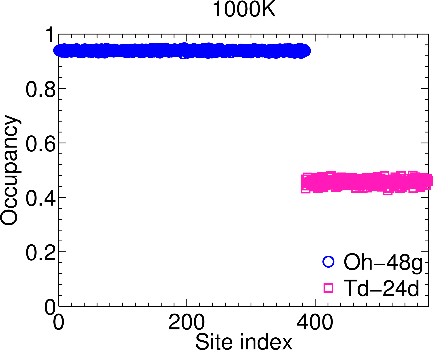 |
| (j) | (k) |
| 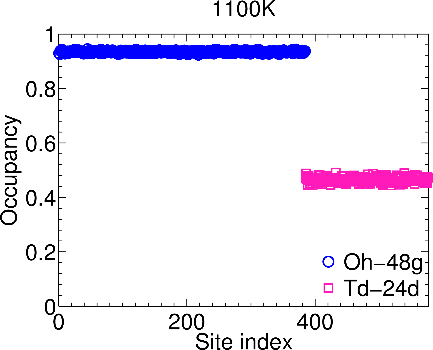 | 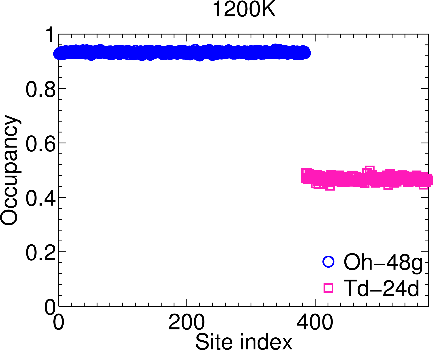 |

Figure S7. Site occupancy with temperature (a). Individual site occupancy at different temperatures, b-k.

1. **Jump statistics**

The overall jump statistics are shown in Figure S8 for c-LLZO (a) and t-LLZO (b)

| (a) | (b) |
| --- | --- |
| 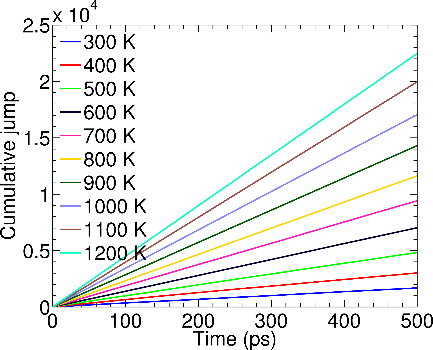 | 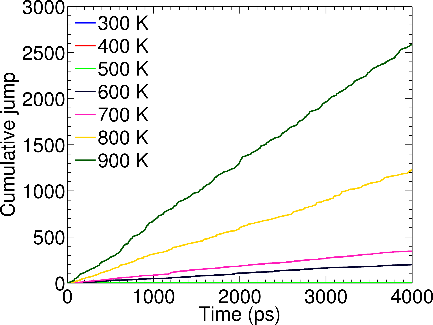 |

Figure S8. Cumulative jump per unit cell with time for c-LLZO (a) and t-LLZO (b).

Jump per unit cell every ps is shown versus temperature in Figure S9.


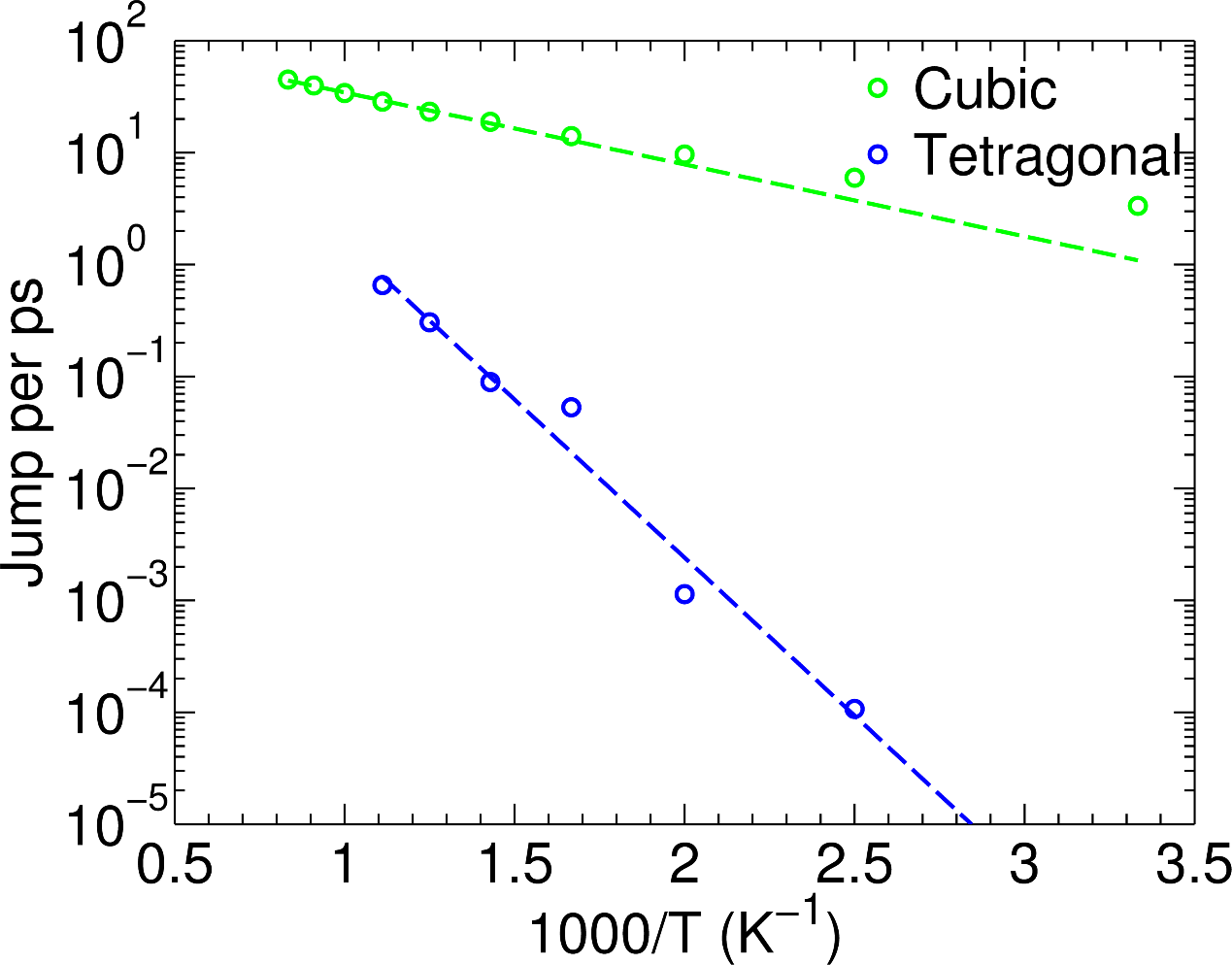


Figure S9. Jump per ps for a unit cell in the cubic cell versus tetragonal cell.

We notice that some Li jumps back to the original sites after leaving this site, as shown in Figure S10.

| (a) | (b) |
| --- | --- |
| 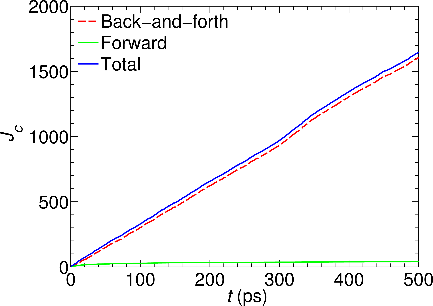 | 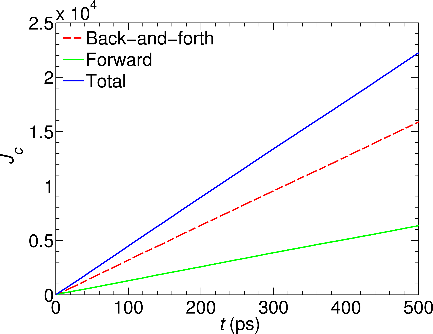 |

Figure S10. One example of the discrimination between forward jump and back-and-forth jump at 300K (a) and 1200K in c-LLZO.

We notice that for most occasions, Li jumps from one site to anther and then jump back in succession steps. This types of jump induces no change for Li diffusion and therefore does not contribute to the Li diffusion. The distribution of traveled distance of jumps and vibration is shown in Figure S11.

| (a) | (b) | (c) |
| --- | --- | --- |
| 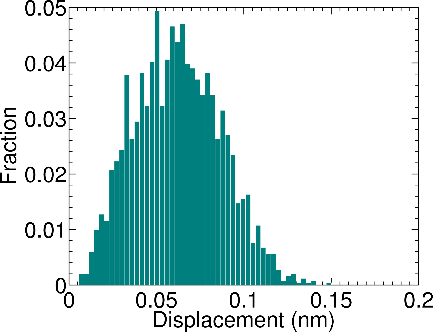 | 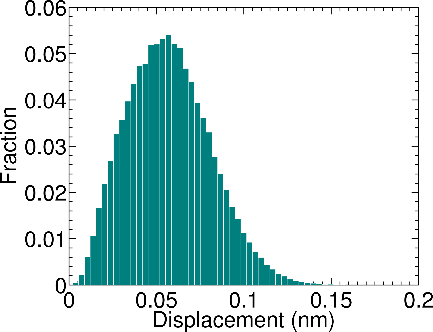 | 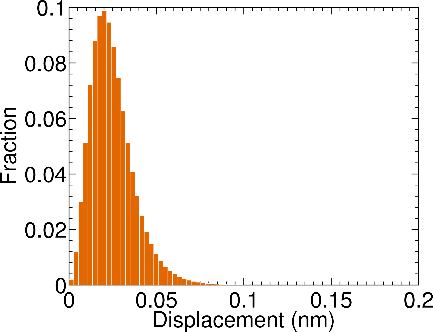 |

Figure S11. Li travel distance distribution, forward jump events (a), back-and-forth jump events (b) and oscillation (c).

The back-and-forth type of jumps are different from the vibration, as shown in Figure S11b and S11c.

| (a) | (b) |
| --- | --- |
| 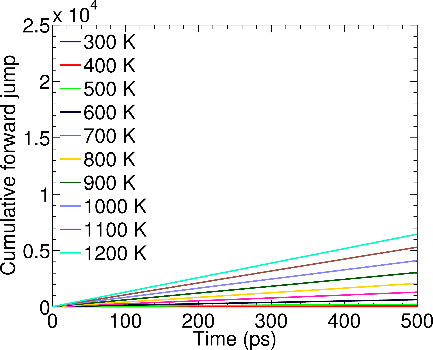 | 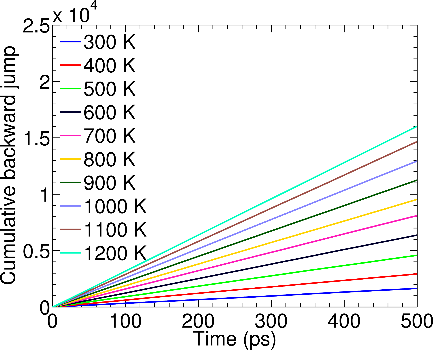 |
| (c) | (d) |
| 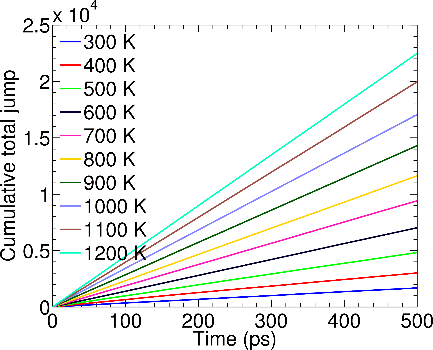 | 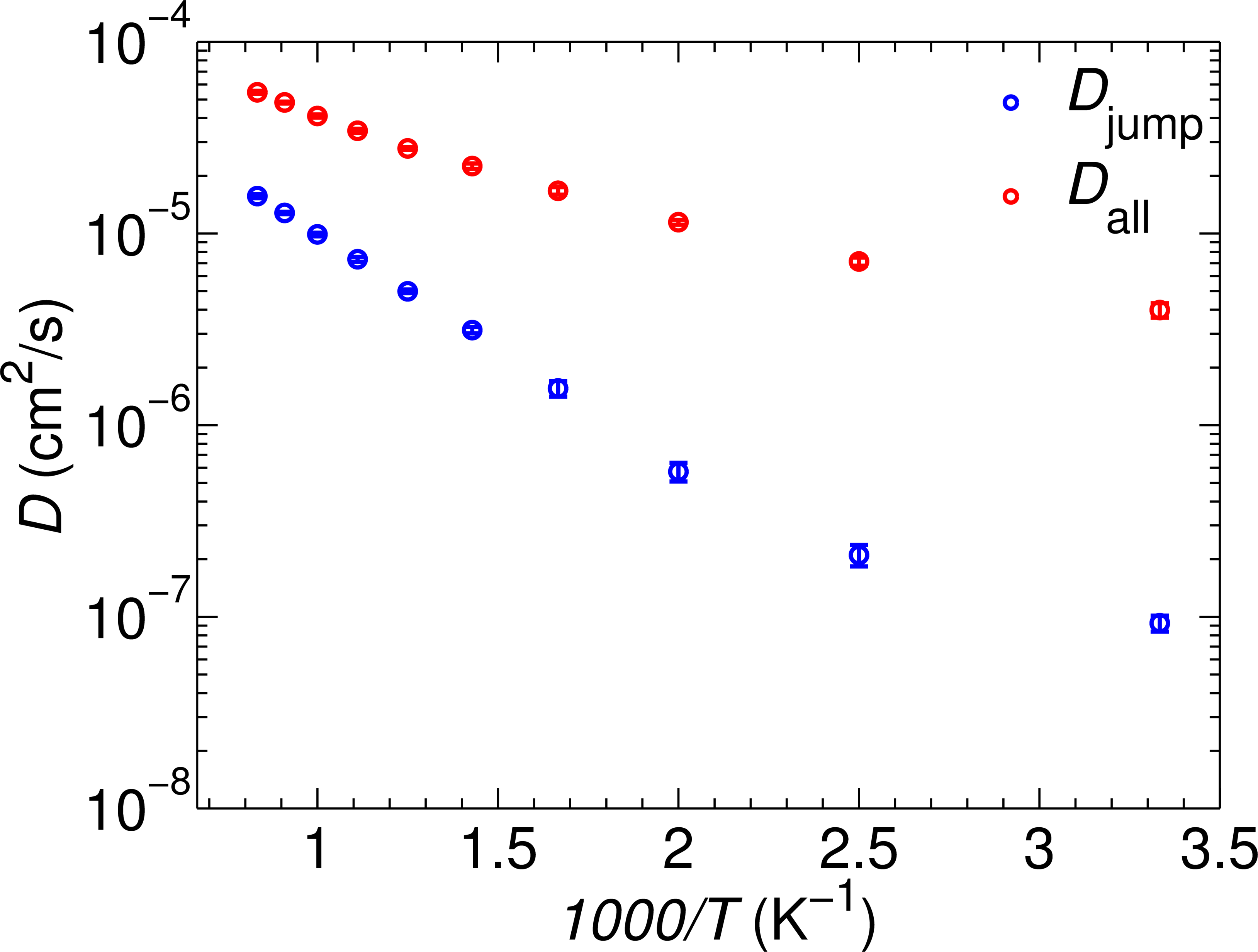 |

Figure S12. Cumulative forward jump times for a unit cell (a), analogously for backward jump (b) and total jump (c). The jump diffusivity calculated from forward jump D_jump_ and total jump D_all_ (d).

| (a) | (b) |
| --- | --- |
| 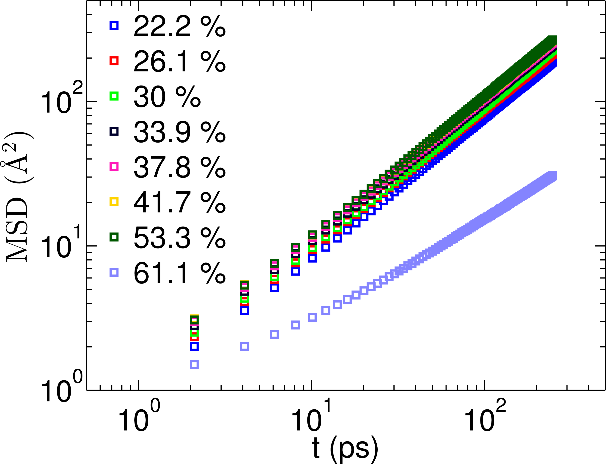 | 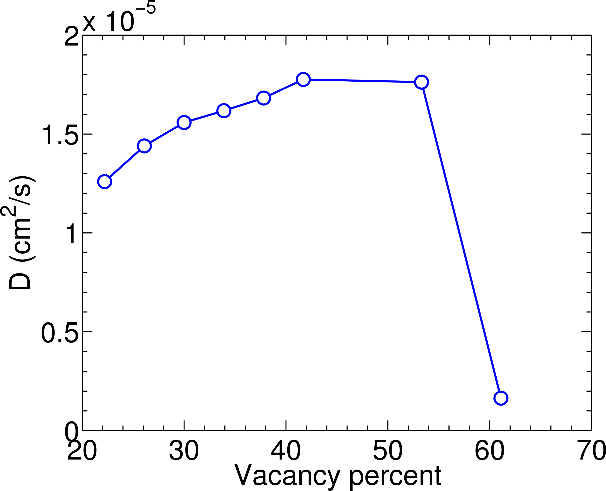 |

Figure S13. The MSD of Li in c-LLZO with vacancy percent x in Li_9-9x_La_3_Zr_2_O_12_ from 22.2% (Li_7_La_3_Zr_2_O_12_) to 61.1% (a) and the corresponding self diffusivity (b).

The jump statistics vs ideal uncorrelated Poisson process is shown in Figure S13 for cubic and tetragonal phases.

c-LLZO

| 300 K | 400 K |
| --- | --- |
| 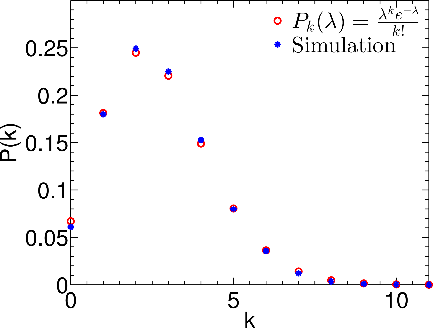 | 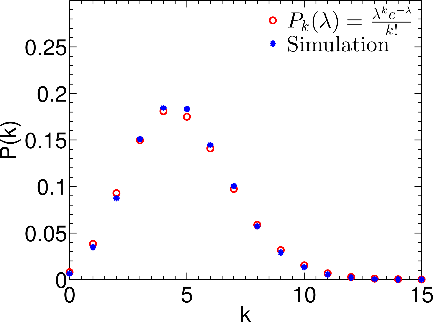 |
| 500 K | 600 K |
| 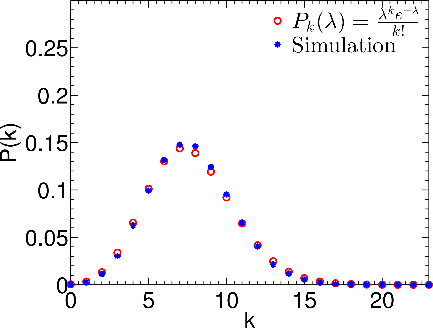 | 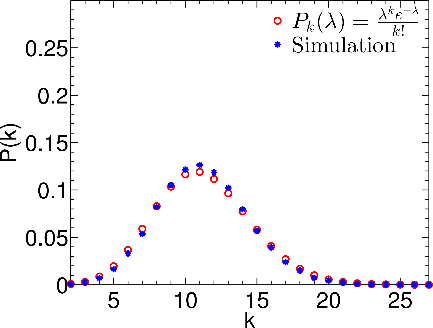 |
| 700 K | 800 K |
| 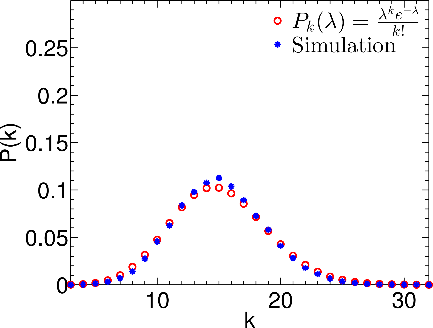 | 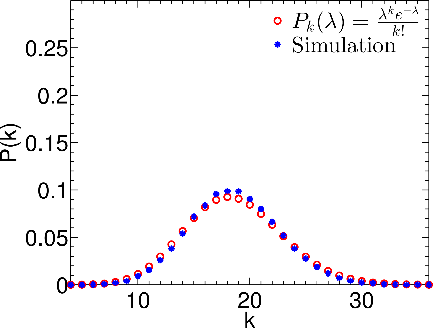 |
| 900 K | 1000 K |
| 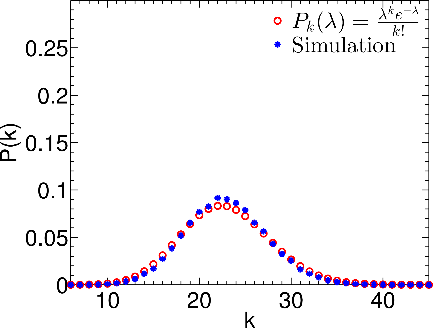 | 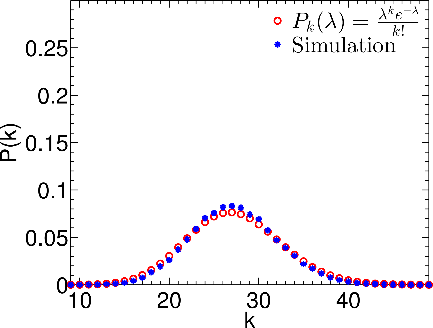 |
| 1100 K | 1200 K |
| 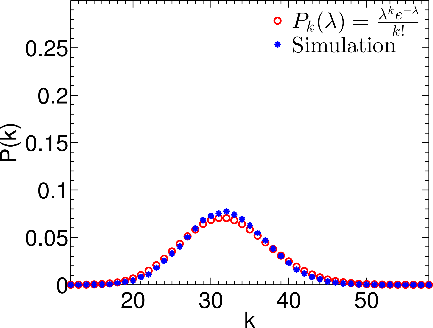 | 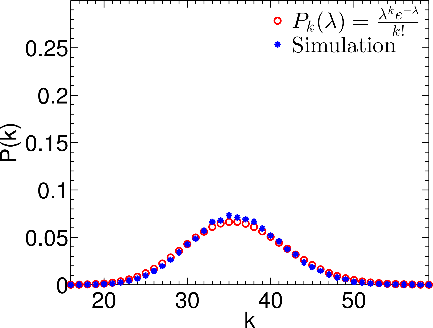 |

t-LLZO

| 600 K | 700 K |
| --- | --- |
| 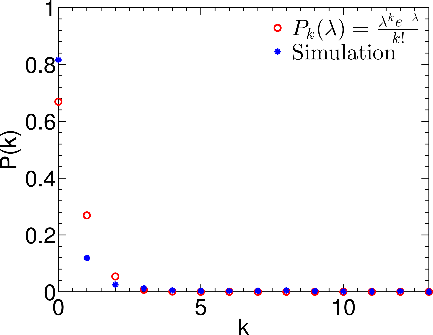 | 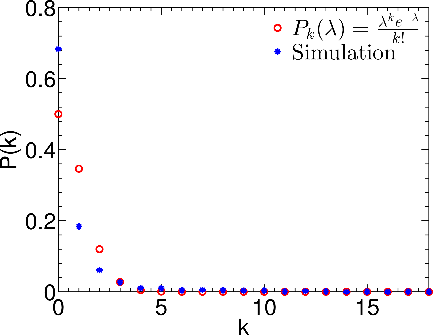 |
| 800 K | 900 K |
| 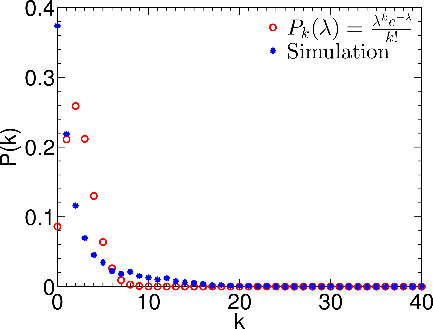 | 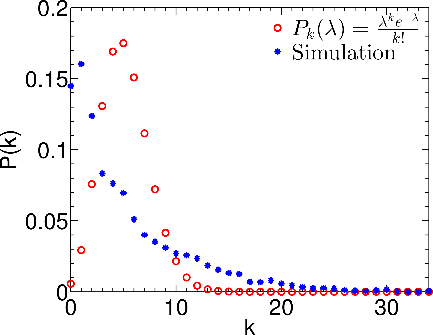 |
|  |  |
|  |  |

Figure S14. Jump statistics from simulations versus Poisson process.

Only temperatures from 500 K to 900 K are shown for t-LLZO, because below 500 K, the jump events happen rarely and above 900 K, phase transition happens.

We used the Cramér-Von Mises goodness-of-fit statistics to quantitatively verify the independent hopping assumption and whether the jump statistics follow a Poisson distribution [^6^](#_ENREF_6).

| 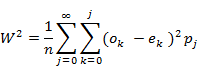 | (2) |
| --- | --- |

Where n is the total number of observations, in our case which we split sample the MD trajectory at 0.1 ps interval for 500 ps, n = 5000,
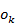
 is the number of times within n that k events happen, and
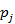
 is the probability from the distribution function that j events happen within the time interval and
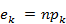
, is the expected events.

Typically, a smaller value of
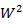
 indicates better goodness-of-fit. In c-LLZO, we plot the
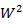
 statistics with respect to temperature, as shown below,

| (a) | (b) |
| --- | --- |
| 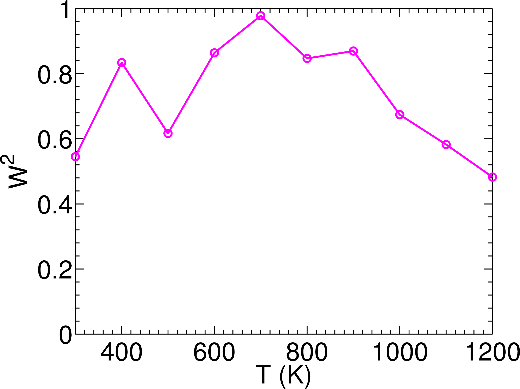 | 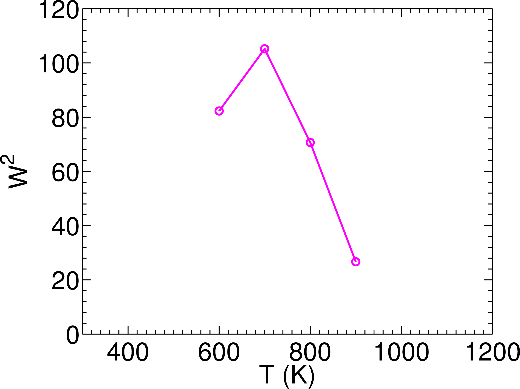 |

Figure S15. The
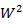
 versus temperatures for cubic (a) and tetragonal (b) LLZO.

As shown in Figure S14, the
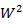
 are smaller than 1 for c-LLZO while these values for t-LLZO are much greater. These results show that the Li hopping in the cubic phase is mostly independent while the tetragonal structure shows correlated jumps. In the cubic case, we notice that temperature below 600 K gives limited number of Li jump, therefore, the statistics is not reliable and the data is omitted from the plot. At temperature greater than 900 K, phase transition occurs and the transporting mechanism becomes identical to the cubic. This is also revealed from the MSD and diffusivity plot in Figure S2.

By changing the sampling rate, the obtained statistics change slightly but this does not affect the conclusion, as shown in Figure S15.

| (a) | (b) |
| --- | --- |
| 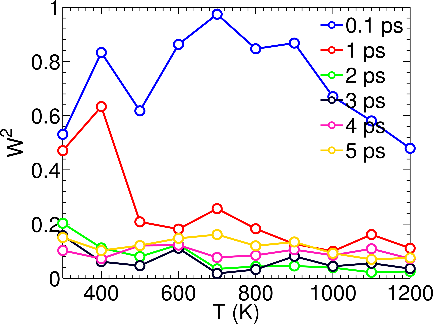 | 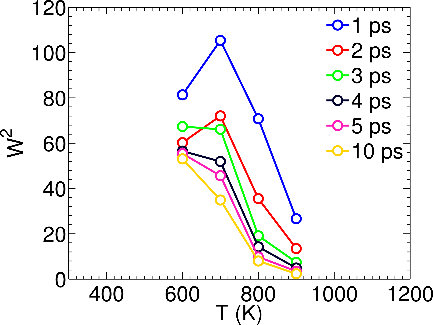 |

Figure S16. *W^2^* change with sampling rate and temperature and the conclusions are the same.

1. **Mutual information calculation**

The entropy is computed as follows

| 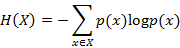 | (3) |
| --- | --- |

where the probability p(x) is defined for one site x as the fraction of occupied time in the total time period.

The mutual information is computed by the following equation

| 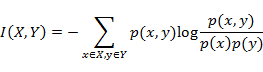 | (4) |
| --- | --- |

X and Y are the occupancies of the two sites, the distribution on the site is a binary distribution, with 1 indicating occupancy and 0 non-occupancy. The mutual information is related to the correlation between the sites. Notice that the advantage of mutual information compared to the correlation matrix is that the mutual information is not limited to real numbers.

| (a) | (b) | (c) |
| --- | --- | --- |
| 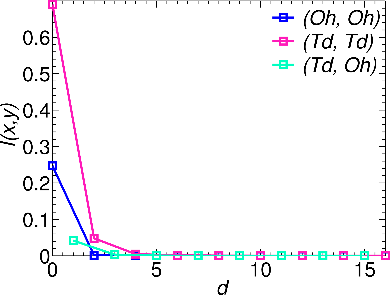 | 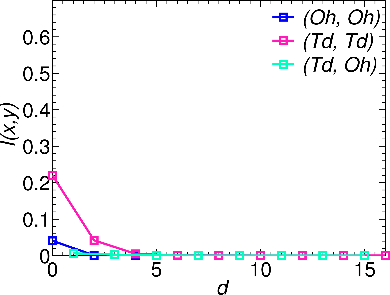 | 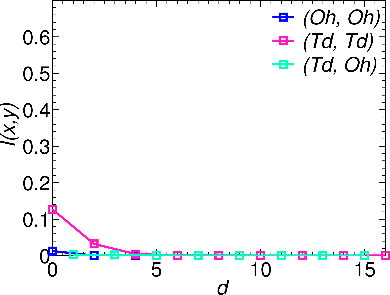 |

Figure S17. Mutual information as function of site separation d, for delay = 0 (a), delay = 0.1 ps (b) and delay = 0.2 ps (c).

The mutual information as a function of spatial separation and time delay at different temperatures are shown below.

| (a) |
| --- |
| 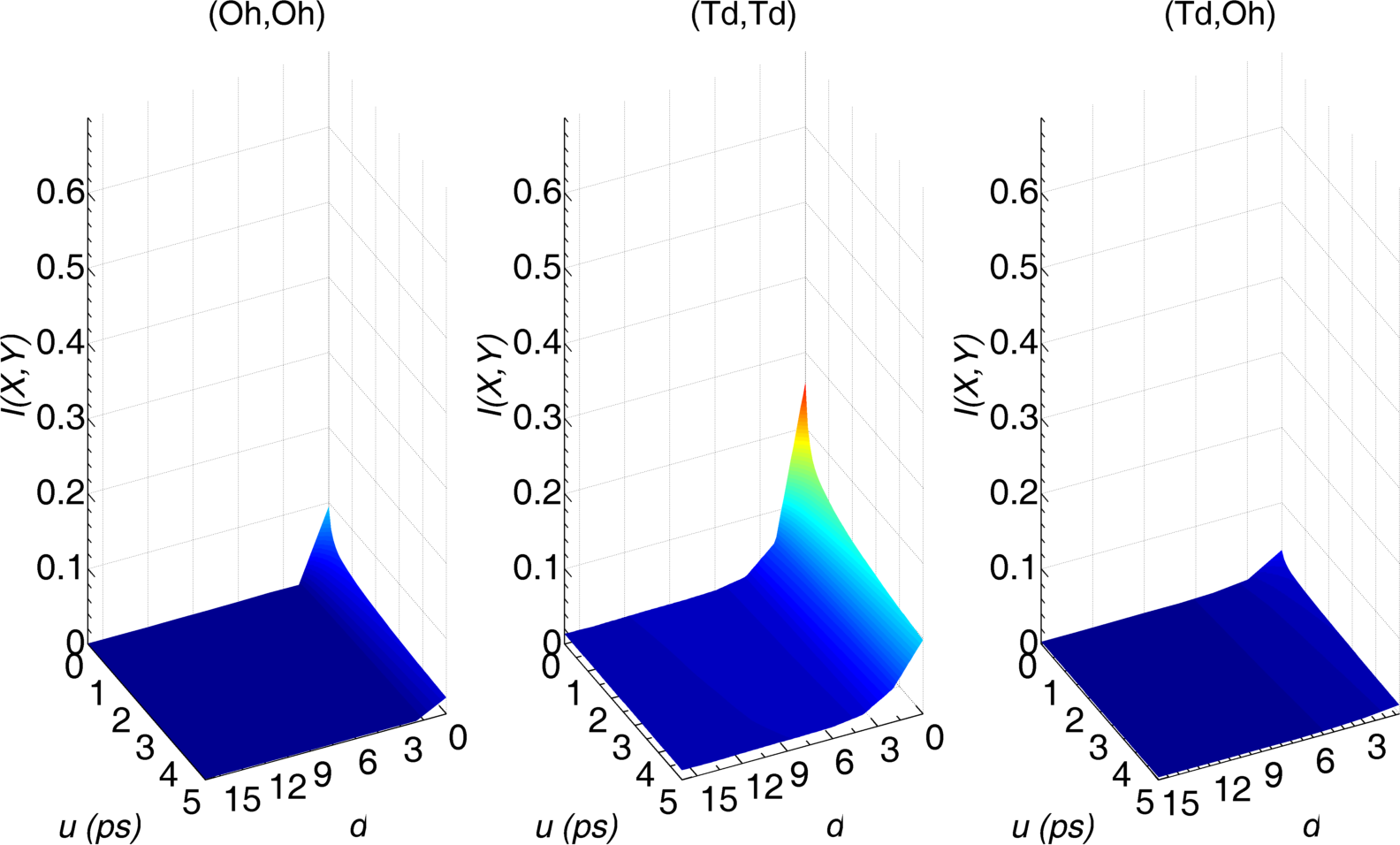 |
| (b) |
| 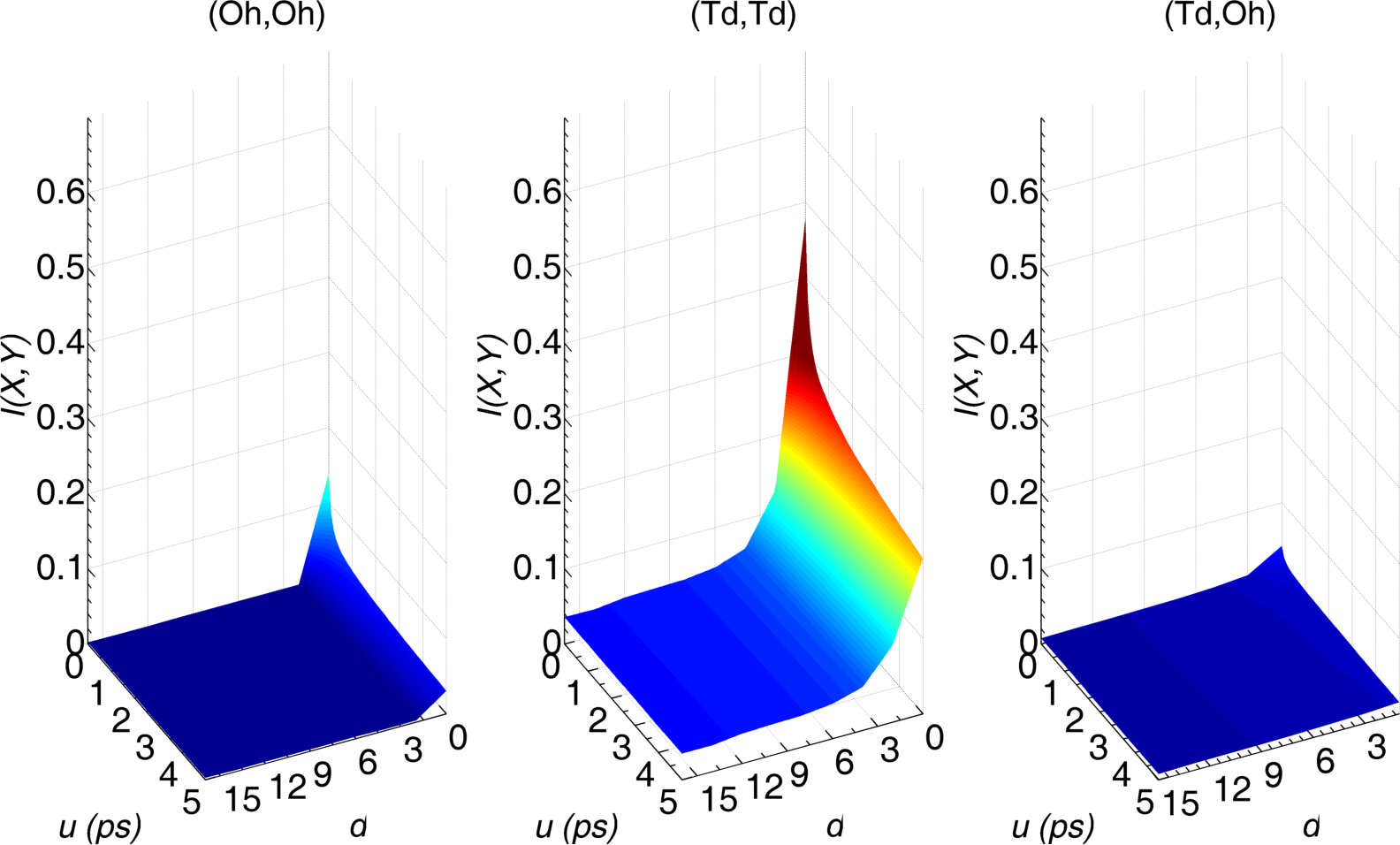 |
| (c) |
| 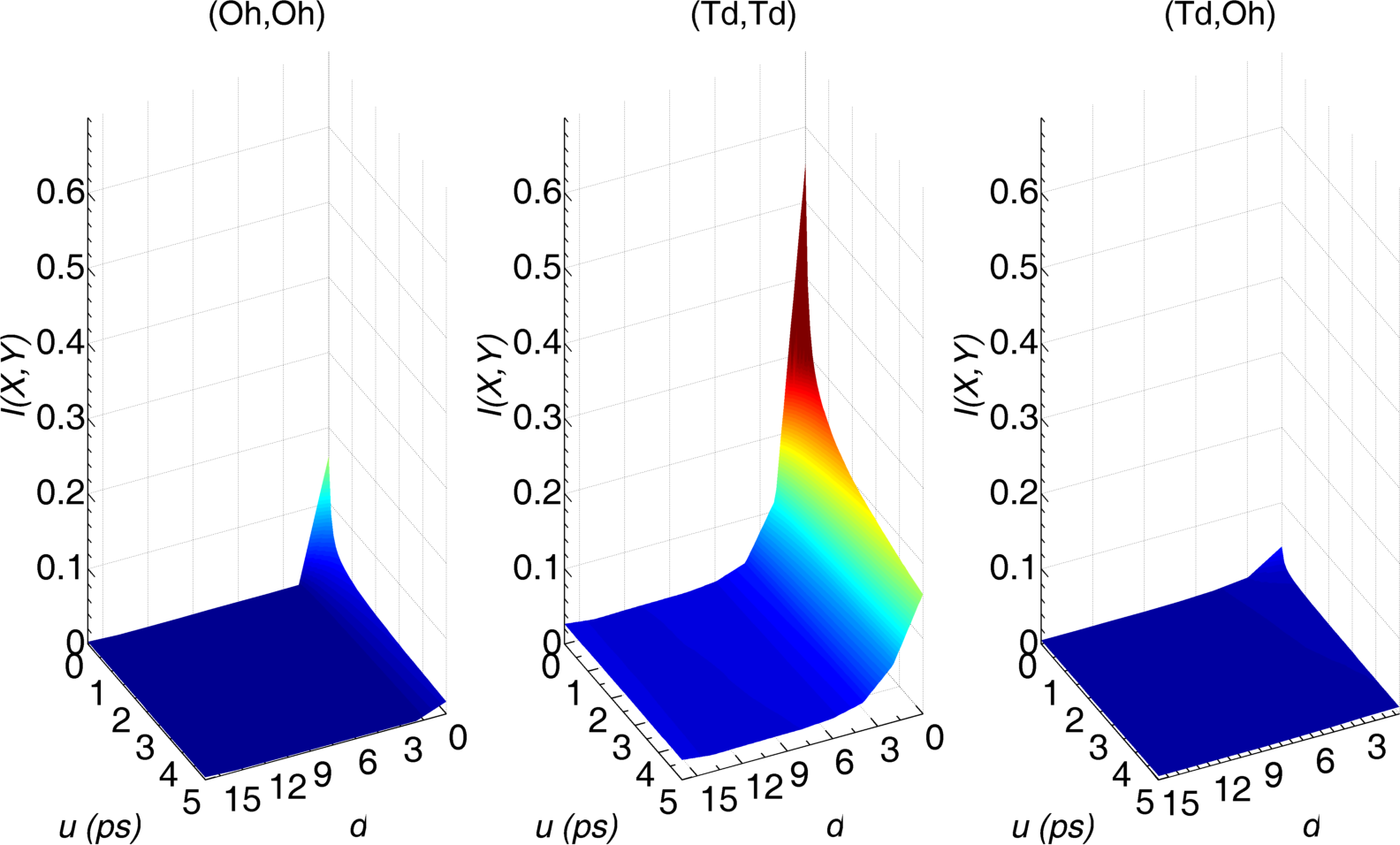 |
| (d) |
| 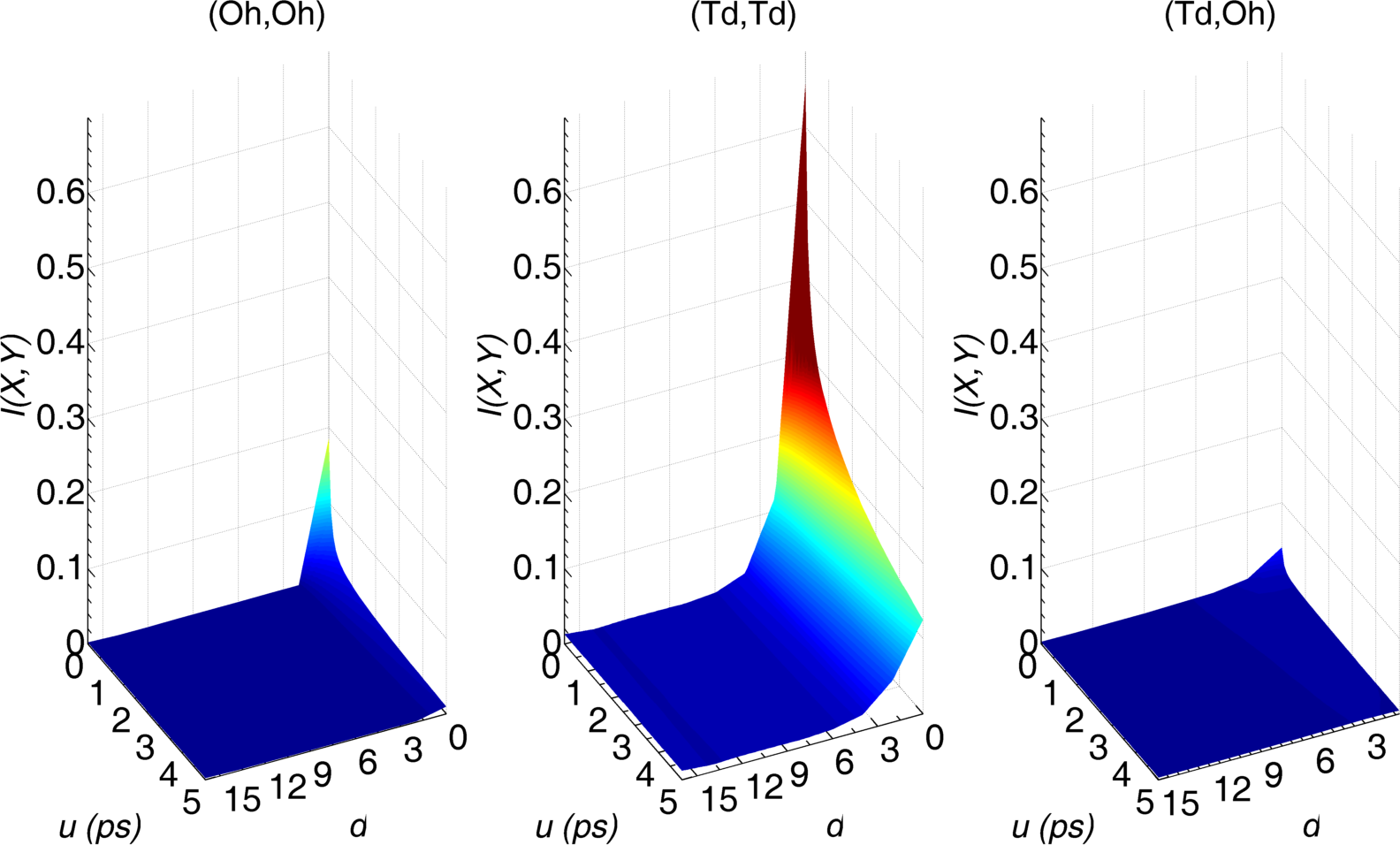 |
| (e) |
| 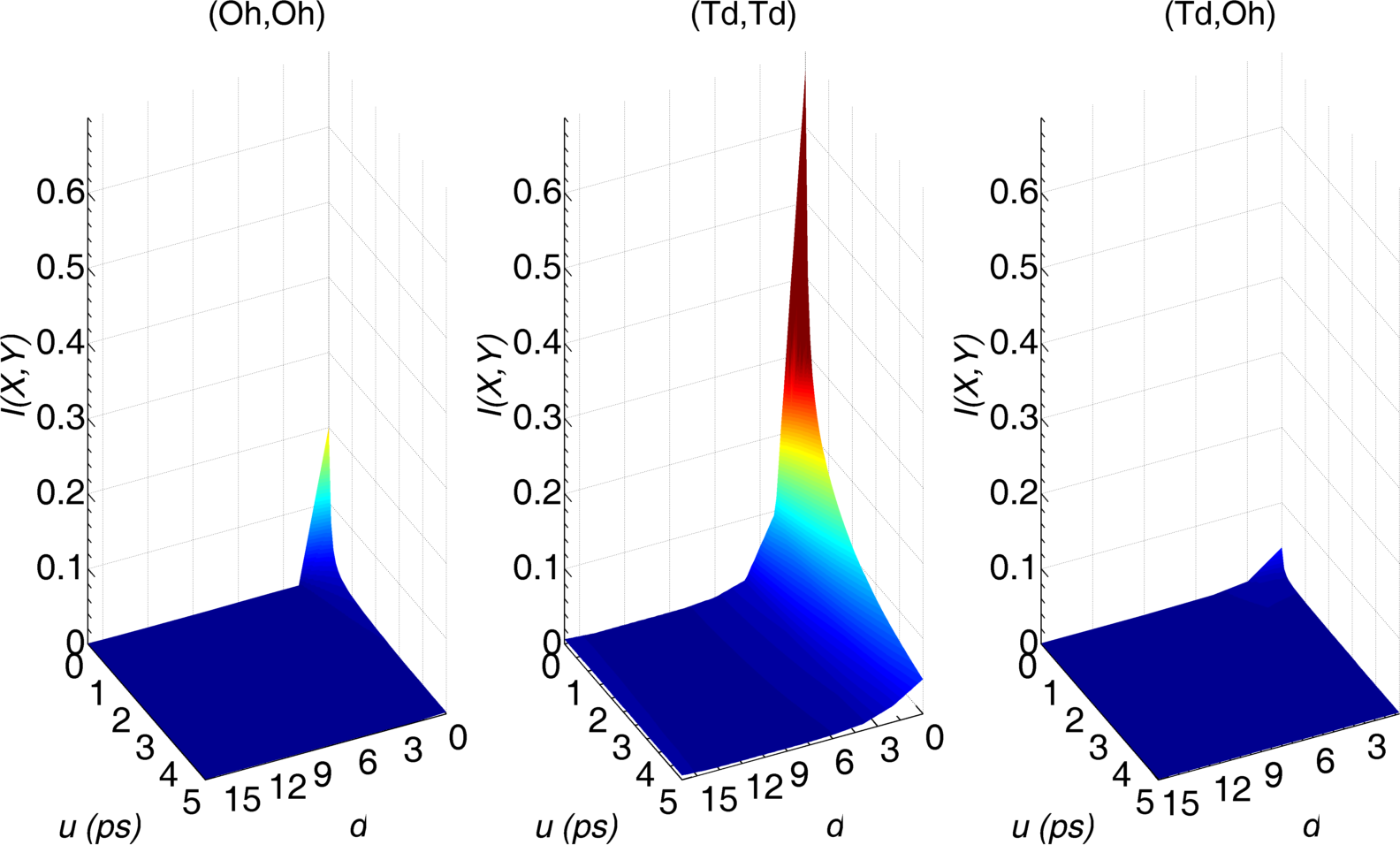 |
| (f) |
| 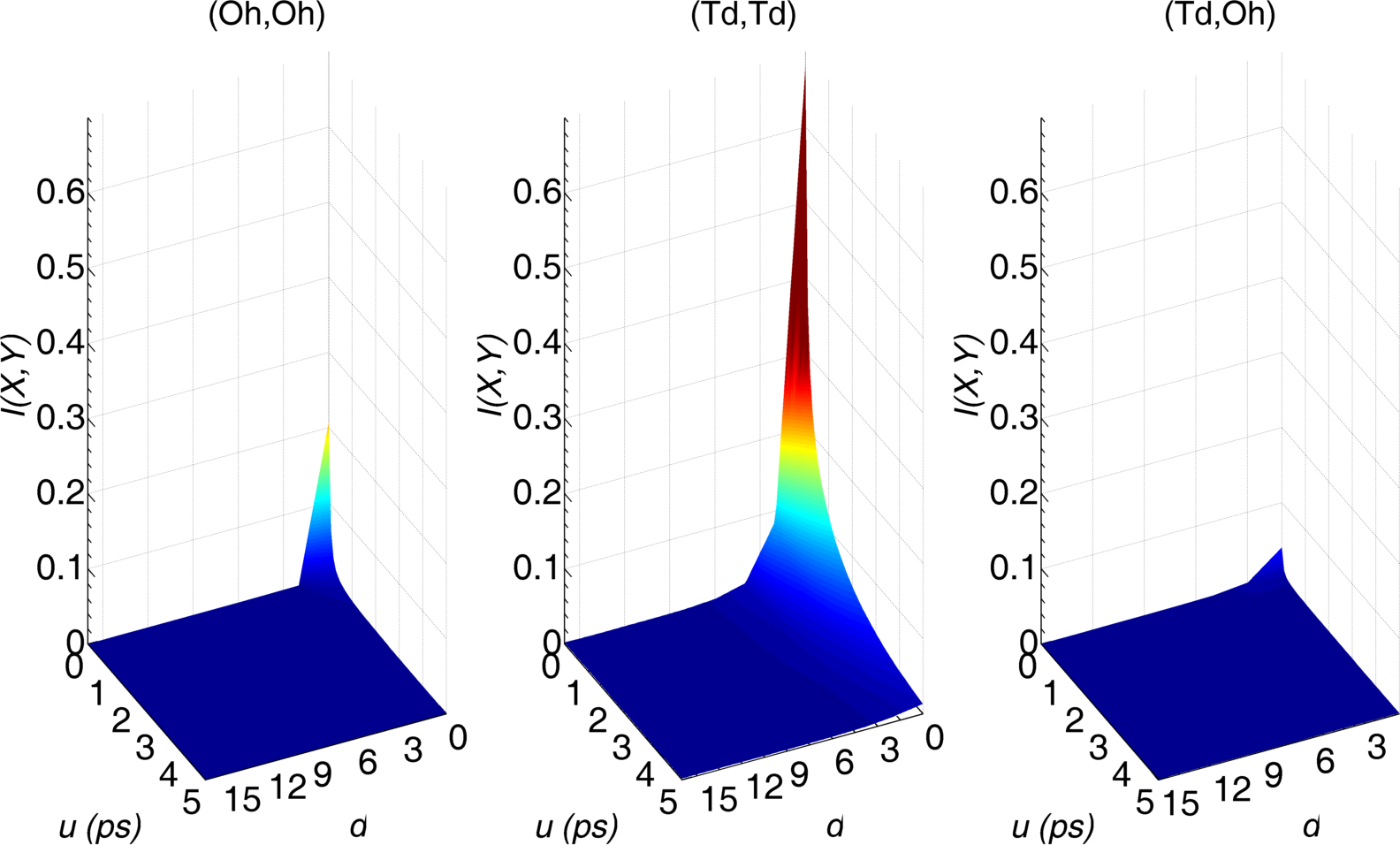 |
| (g) |
| 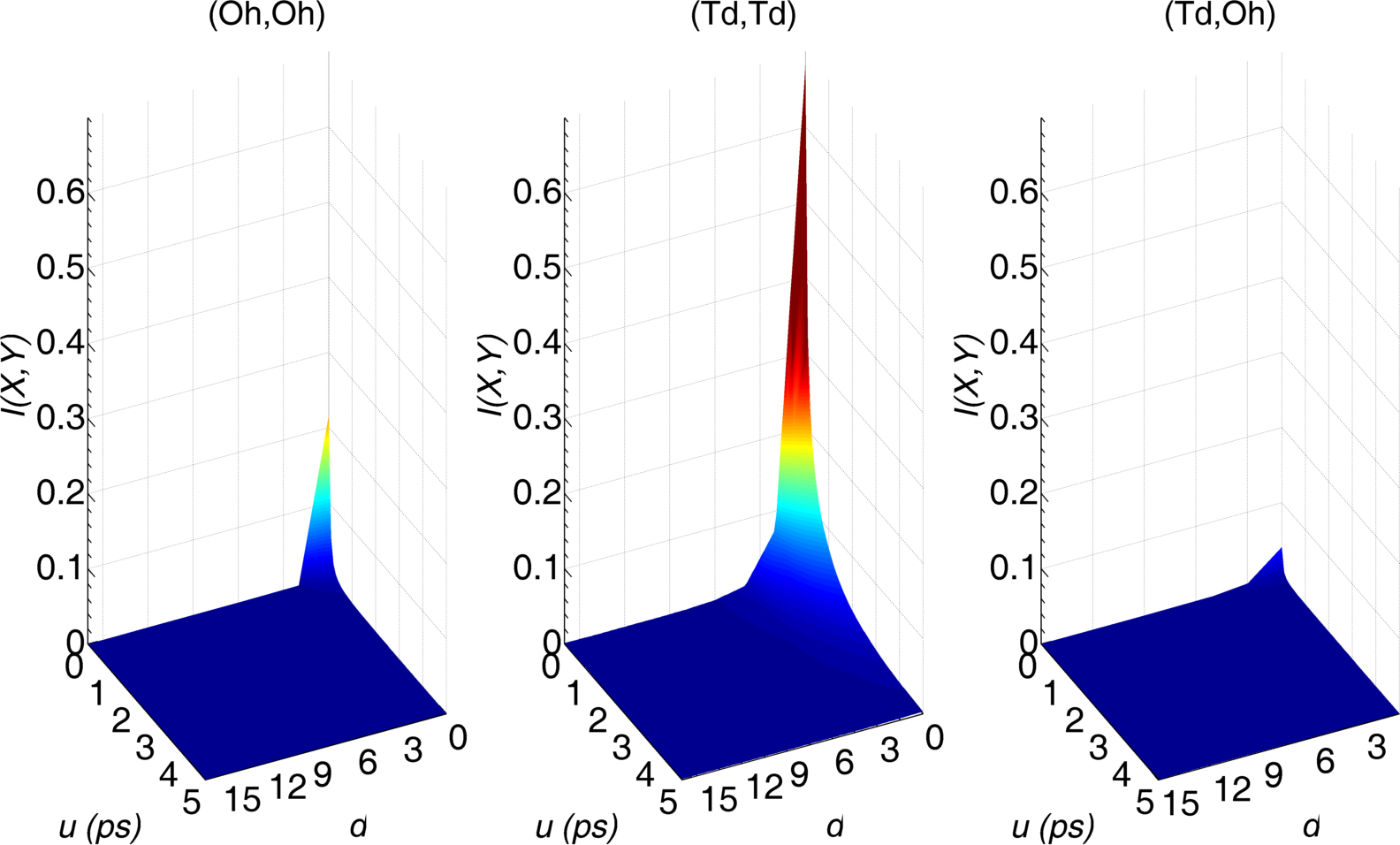 |
| (h) |
| 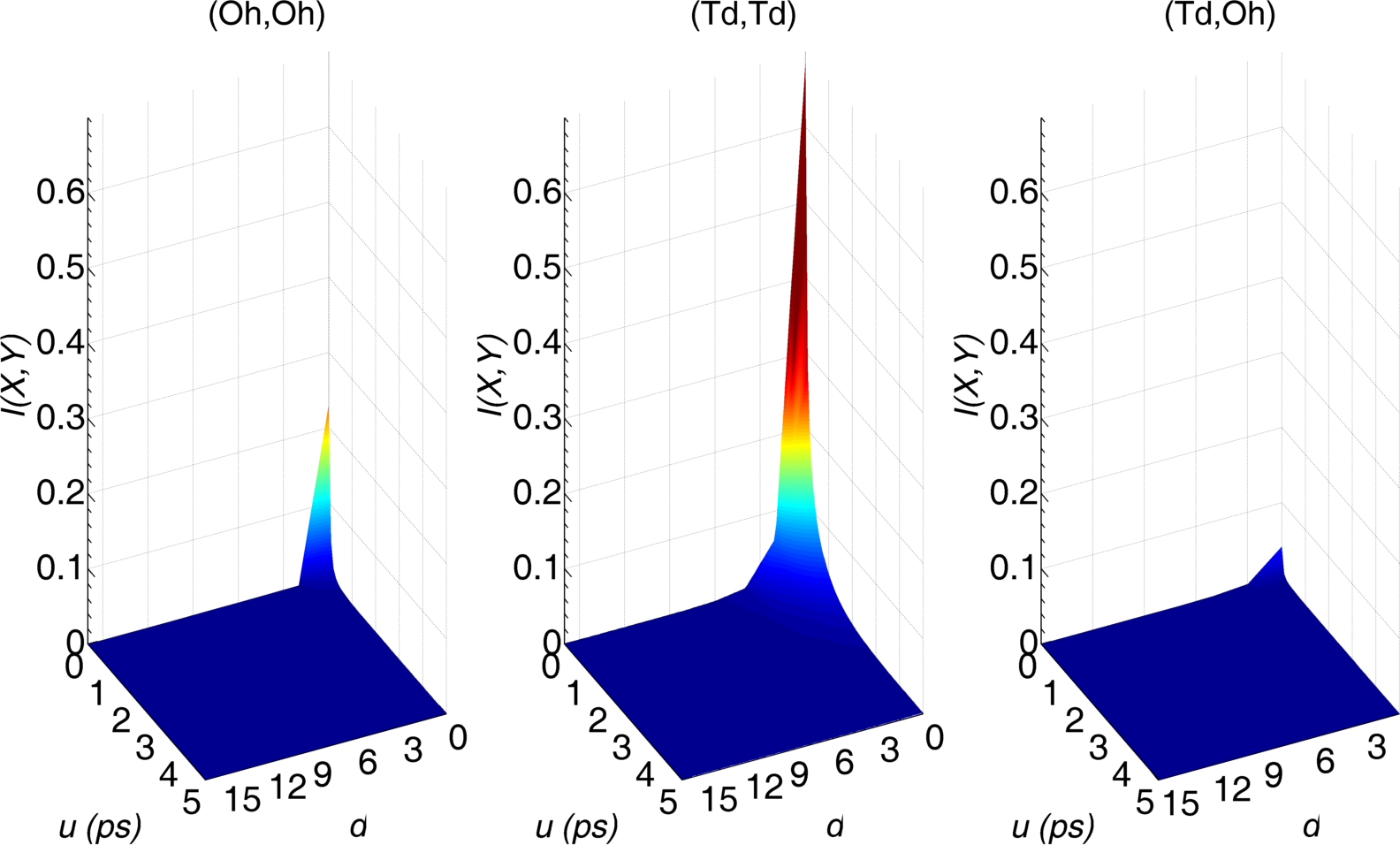 |
| (i) |
| 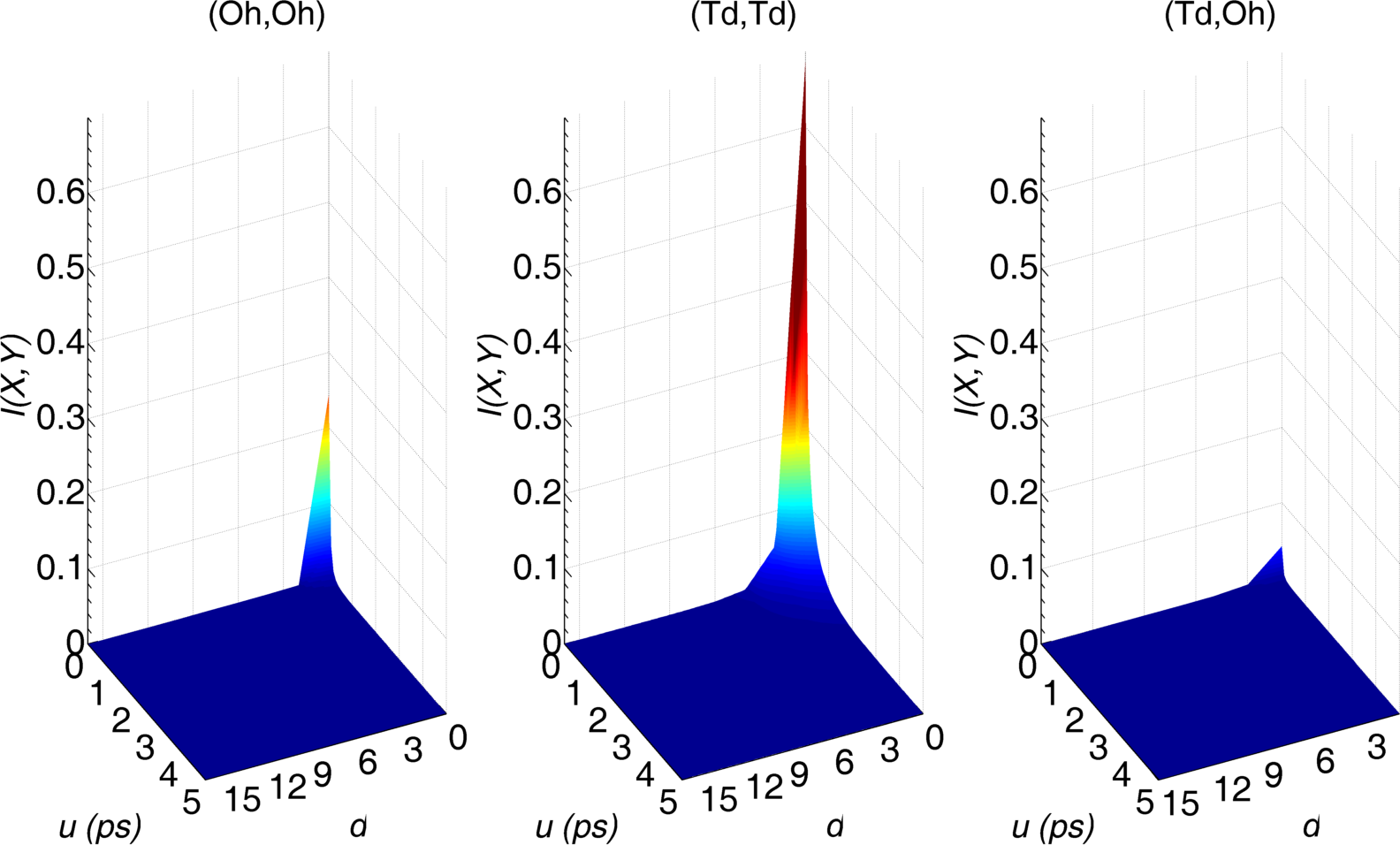 |
| (j) |
| 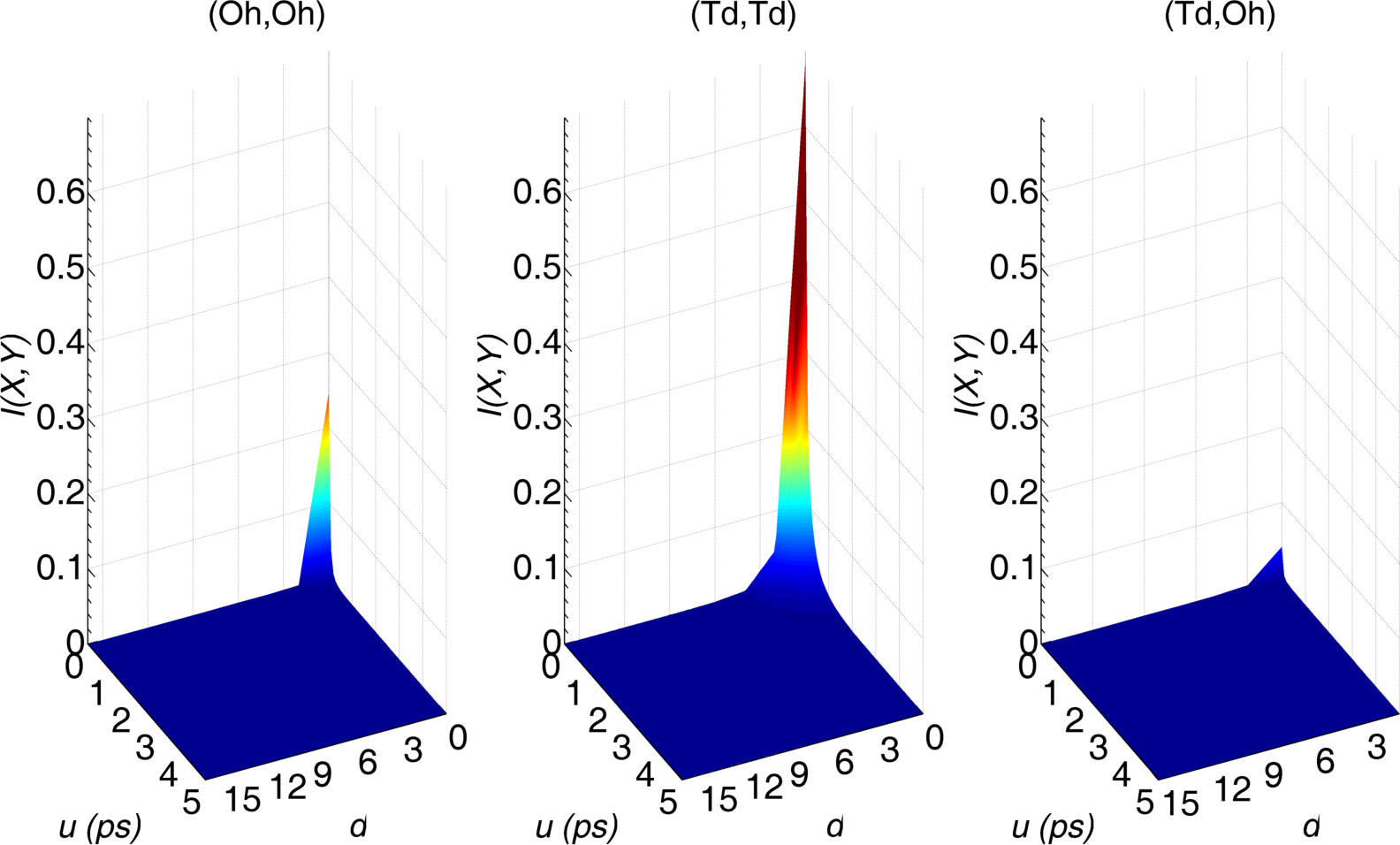 |

Figure S18. (a) to (j) correspond to the mutual information with time delay and site distance separation at temperature from 300 K to 1200 K with 100 K interval.

From Figure S18, one observation is that with increasing temperature, the entropy for both Td and Oh site occupancy increases as expected from greater randomness. On the other hand, the autocorrelation (distance = 0) decreases faster at higher temperature, indicating a faster dynamics. Overall, the neighbor interaction is weak, explaining the observed Poissonian type of diffusion, as shown in the (Td, Oh) panels. The interaction is also short-ranged in space, and the mutual information decays faster with distance at higher temperature as well.

**References**

1 Klenk, M. J. & Lai, W. Finite-size effects on the molecular dynamics simulation of fast-ion conductors: A case study of lithium garnet oxide Li_7_La_3_Zr_2_O_12_. *Solid State Ionics* **289**, 143-149 (2016).

2 Wang, Y., Klenk, M., Page, K. & Lai, W. Local Structure and Dynamics of Lithium Garnet Ionic Conductors: A Model Material Li_5_La_3_Ta_2_O_12_. *Chem. Mater.* **26**, 5613-5624 (2014).

3 Klenk, M. & Lai, W. Local structure and dynamics of lithium garnet ionic conductors: tetragonal and cubic Li_7_La_3_Zr_2_O_12_. *Phys. Chem. Chem. Phys.* **17**, 8758-8768 (2015).

4 Skiena, S. Dijkstra's algorithm. *Implementing Discrete Mathematics: Combinatorics and Graph Theory with Mathematica, Reading, MA: Addison-Wesley*, 225-227 (1990).

5 Chen, D. *et al.* Compositional engineering of perovskite oxides for highly efficient oxygen reduction reactions. *ACS Appl. Mater. Interfaces* **7**, 8562-8571 (2015).

6 Morgan, B. J. & Madden, P. A. Relationships between atomic diffusion mechanisms and ensemble transport coefficients in crystalline polymorphs. *Phys. Rev. Lett.* **112**, 145901 (2014).
